# Review of nanomaterial advances for ionizing radiation dosimetry

**DOI:** 10.1063/5.0134982

**Published:** 2023-06

**Authors:** Eslam Aboelezz, Brian W. Pogue

**Affiliations:** 1 Ionizing Radiation Metrology Department, National Institute of Standards, Giza, Egypt; 2 Department of Medical Physics, University of Wisconsin-Madison, Madison 53705, USA

## Abstract

There are a wide range of applications with ionizing radiation and a common theme throughout these is that accurate dosimetry is usually required, although many newer demands are provided by improved features in higher range, multi-spectral and particle type detected. Today, the array of dosimeters includes both offline and online tools, such as gel dosimeters, thermoluminescence (TL), scintillators, optically stimulated luminescence (OSL), radiochromic polymeric films, gels, ionization chambers, colorimetry, and electron spin resonance (ESR) measurement systems. Several future nanocomposite features and interpretation of their substantial behaviors are discussed that can lead to improvements in specific features, such as (1) lower sensitivity range, (2) less saturation at high range, (3) overall increased dynamic range, (4) superior linearity, (5) linear energy transfer and energy independence, (6) lower cost, (7) higher ease of use, and (8) improved tissue equivalence. Nanophase versions of TL and ESR dosimeters and scintillators each have potential for higher range of linearity, sometimes due to superior charge transfer to the trapping center. Both OSL and ESR detection of nanomaterials can have increased dose sensitivity because of their higher readout sensitivity with nanoscale sensing. New nanocrystalline scintillators, such as perovskite, have fundamentally important advantages in sensitivity and purposeful design for key new applications. Nanoparticle plasmon coupled sensors doped within a lower Z_eff_ material have been an effective way to achieve enhanced sensitivity of many dosimetry systems while still achieving tissue equivalency. These nanomaterial processing techniques and unique combinations of them are key steps that lead to the advanced features. Each must be realized through industrial production and quality control with packaging into dosimetry systems that maximize stability and reproducibility. Ultimately, recommendations for future work in this field of radiation dosimetry were summarized throughout the review.

## I. INTRODUCTION

Radiation fields are often detected directly using ionization chambers and semiconductor sensors, as well as passively using optically stimulated luminescence (OSL), radiochromic film, thermoluminescence (TL), Fricke gel (FG), and electron spin resonance (ESR), each of which is composed of nanoscale materials that interact with the radiation dose in unique ways. The specific component materials used to create each device varies widely, and the choices developed for these alter the performance of the measurements possible. Thus, this review focuses on the nanolevel material composition of these basic components in dosimetry and highlights the emerging features of each.

### A. Radiation dosimeters

Physical, chemical, or electronic radiation dosimeters are designed to measure or assess the radiation quantities: absorbed dose in media (water, air or tissue), exposure, ambient dose, or equivalent dose or their rates. Such dosimeters in combination with a specific reader form a given dosimetry system. In addition to accuracy and precision, the essential characteristics of any dosimeter system includes range of linearity, energy dependence, dose response characterization, dose rate dependence, angular response, temporal response, post-irradiation effect, and spatial resolution needed and possible.^1^ However, there is no dosimeter that can fulfill all desired properties and the choice of the dosimeters relies upon the circumstances and requirements of the measurements. As a result, ongoing research into new dosimeters or the development of well-known established dosimeters with improved features is critical.

Radiation detectors based on storage phosphors are the most used conventional passive dosimeters because they have the ability to record the radiation dose and how it is distributed in the absorbed medium. The primary sensing element of these kinds of dosimeters is a storage phosphor that records accumulated dose over the period of use. When the storage phosphor absorbs the energy from ionizing radiation, a significant number of charge carrier pairs are formed in the matrix, with the number of pairs being proportional to the radiation dose. The radiation-induced carriers are then captured and kept localized in trapping centers until stimulated and become freely moving, they then recombine at the luminescence center, producing photons with a wavelength range from ultraviolet (UV) to near-infrared (NIR). The form of this stimulation can be heat or light usually visible or NIR, and the associated luminescence is referred to as TL^2^ or OSL,^3^ respectively. Localized trapping centers containing trapped electrons or holes can act as luminescence centers in some exceptional cases, and this phenomenon is known as radio-photoluminescence (RPL).^4^ In many circumstances, RPL is caused by a modification in the valence state of an impurity ion (e.g., 2Ag^+^ → Ag^0^ + Ag^2+^), and in other cases, RPL is generated by a defect-type luminescence F-center. Because the emission intensity, which refers to the number of emitted photons resulting from TL, OSL, and RPL, is related to the absorbed radiation dose, the emission intensity can be used to readout and indirectly estimate the radiation dose.

Apart from the material structure, it is generally thought that the number of discovered materials with storing luminescence capabilities are roughly ranked as RPL > OSL > TL in order of difficulty and commonality. Among synthesized novel materials, it was found that many of them (>90%) have quantifiable TL and almost half of them have OSL response. On the other hand, RPL material is uncommon, with less than 5% of materials synthesized being RPL.^5^ Molecular and nanoscale sensors can transcend the constraints of traditional systems and are facile and efficient as radiation sensors.^6^

### B. Nanomaterials

Particles with dimension size ranging from 1 to 100 nm are referred to as nanoparticles (NPs), and these include nanopowders, pressed compacts, films, and nanoceramic materials. There is significant interest in them because they are applicable in several research areas, including the food industry,^7^ materials science,^8^ and the majority of current health applications.^9,10^ Their major attractive features are their exclusive, distinct, and/or multifunctional properties. An important feature of NPs is the ability to adjust their geometrical properties through varying their shape, size, size distribution, and surface properties and production technique, each of which can alter their performance in dose recording and readout. Their physicochemical features are altered by variations in geometry, affecting photochemical, magnetic, phosphorescence, or fluorescence features. Most interestingly, the nanomaterial versions of materials, in many instances, act differently from their bulk counterparts,^11^ and so study and attention of the NP features is an important part of the science of dosimetry.

Powder nanophosphors with particle sizes from 1 to 10 nm are challenging to manufacture and store. Such powders are susceptible to agglomeration and, thus, are unstable. Nanophosphors with a particle size of 20–100 nm are easy to utilize. One of the essential features of nanophosphors, at numerous nanograin boundaries, is the high concentration of both surface atoms and defects. Surface trapping centers of charge-carriers, like bulk centers yet with a different energy depth, are created. The breakdown of translational symmetry and the restriction of electron free paths by nanoparticle size affect the selection rules, introduce new optical transitions, raise oscillator power, and vary the luminescence decay time. Quantum confinement becomes effective when the nanoparticle size approaches that of the de Broglie wavelength or the Bohr exciton size, affecting the forbidden gap width and resulting in the creation of new energy levels.^12^

Numerous methods are used to characterize nanomaterials, including atomic force microscopy (AFM) for geometrical research, transmission electron microscopy (TEM), scanning electron microscopy (SEM), dynamic light scattering (DLS) for hydrodynamic size, x-ray diffraction (XRD) for crystalline structure, and energy dispersive x-ray analysis (EDAX) for elemental composition.^13^ In addition, vibrating sample magnetometer (VSM), Fourier transform infrared (FTIR), fluorescence spectroscopy (FL), and UV–visible spectroscopy (UV–Vis) are considered as optional tools helping in the nanomaterials characterization.

### C. Uses of dosimeter nanotechnology in radiotherapy

Radiation dosimetry is a core technology in the treatment of cancer, and the scope of use is truly enormous. Cancer is still one of the largest causes of death globally today, with >18 × 10^6^ new cancer patients and 9.5 × 10^6^ deaths worldwide in 2018, and by 2040, the predicted annual new cancer case rate will reach >29 × 10^6^ with estimated 16 × 10^6^ deaths.^14,15^ Radiotherapy is used to treat nearly half of all cancer patients, both for curative and palliative purposes, as a sole therapeutic modality and in conjunction with other treatments.^16,17^ The measurement and prediction of doses delivered to patients during this therapy is imperative, and high accuracy and precision of delivery needed is critical to ensure that the treatment is more effective than the damage induced by the radiation. Typical doses ranges used are listed in Table I, for radiation therapy and diagnostic imaging. Common practice uses daily fractions of radiotherapy, which is the delivery of a small, prescribed dose in multiple daily sessions, commonly near 1–3 Gy, and given in less than a minute of irradiation. Thus, the dose rate for each session is on the order of Gy/min.^18^

**TABLE I.** Range of doses or effective dose equivalents (EDE) applied in different procedures of radiation therapy and diagnosis. Reproduced with permission from Mosayebi *et al.*, Radiat. Phys. Chem. **164**, 108362 (2019). Copyright 2019 Elsevier.^18^

Imaging or therapy protocol	Dose or effective dose	References
Breast cancer—radiotherapy	40–75 Gy	20
Lung cancer—radiotherapy	45–70 Gy	20
Glioblastoma—radiotherapy	13–90 Gy	20
Pancreas—radiotherapy	45–54 Gy	20
Gene expression	0.7–39 mSv	21
Biodosimetry—high dose	0.5–4 Gy	22
Biodosimetry—low dose	5–100 mGy	22
Cellular experiments	1 mGy-8 Gy	23
Animal experiments	Up to 49 Gy (mice)	24
	68 Gy (rats)	25
Bone scan (Tc-99m)	44 mGy (organ dose) 4.4 mSv (EDE)	26
Thyroid scan (Tc-99m)	13 mGy (organ dose) 2 mSv (EDE)	26
Heart perfusion (Tl-201)	33 mGy (organ dose) 10 mSv (EDE)	26
Tumor (Ga-67)	21 mGy (organ dose) 12 mSv (EDE)	26

Patients can now get effective, practical, inexpensive, and less aggressive cancer treatment thanks to the development of novel and focused cancer therapy procedures^19^ that are validated by high accuracy dosimetry in both pretreatment plan verification to tissue phantoms and with post-treatment verification by on-patient dosimetry tools.

### D. Nanomaterials applications in radiation dosimetry

Nanophosphors have several merits that are significant in radiation detection that are determined by the fundamental attributes listed above. The luminescence output may be increased at high doses owing to the formation of Electron–hole pairs inside the nanoparticles, and separation to their neighbors by a small distance at high doses. As a result, the probability of recombination increases, as does the intensity of the luminescence. Broadening and shifting of the luminescence bands changes in the temperatures at the maximum of the dosimetric peak, and a sharp decrease in the phosphor afterglow period is all known observations.^27^

Nanophosphors have a considerable increase in radiation resistance, although resistance mechanisms are not well established, and the causes are hypothesized. One of these proposed causes is effective defect removal and cancelation at nanograin boundaries, which retards the radiation effect accumulation in nanomaterials.^28^ Furthermore, because the size of a nanoparticle is comparable to the mean free path of electron excitations and the length of radiation defect diffusion, the prompt dissipation of absorbed radiation energy is thought to be due to the effective transition of charge carriers between nearby particles.^27^

Even though nanomaterial dosimetry advances are still evolving, it is conceivable to deduce that nanoparticles can improve and optimize local dose delivery, hence increasing the dosimeter sensitivity. The nanoparticles can also enhance light emission by coupling to radiation-induced luminescent centers. The development of nanoparticle-assisted dosimetry becomes a very exciting, challenging, and new research field because many factors can influence the dosimetric features, such as nanoparticle size, position, and plasmon properties, as well as defect related phenomena arising from the addition of nanoparticles into a given matrix. Developments are motivated by the possible application of metal nanoparticles in scintillation and thermoluminescence, in addition to other ESR, OSL, and TL sensitive materials and nanocomposites with diverse configurations (such as core–shell nanoparticles).^29^ One of the main drawbacks of a potential dosimeter containing high Z_eff_ nanomaterials in the radiation dosimetry applications is the response discrepancy for different beam qualities. This can be overcome by distributing a small amount of nanophosphor in a transparent plastic or liquid with a low Z_eff_ and making the dosimeter tissue-equivalent.^30^

## II. ENHANCEMENT OF VARIOUS RADIATION DOSIMETRY SYSTEMS USING NANOTECHNOLOGY

### A. Thermoluminescence dosimetry

**FIG. 1.**
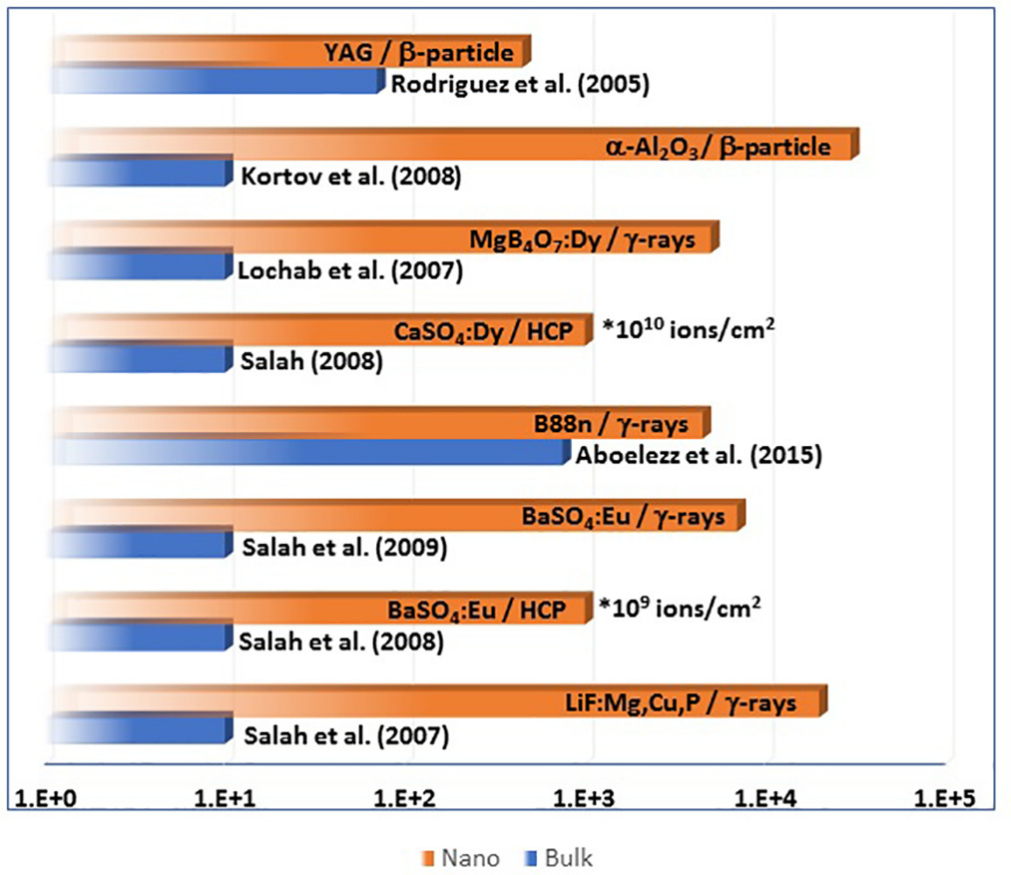
Comparison between the upper detectable limits for some TL dosimeters based on nanoparticle and microparticle (bulk) materials compositions.^36,38–44^ The units of the x axis are Gy for dose or bars are labeled with ions/cm^2^ for fluence of heavy charged particles (HCPs). The α-Al_2_O_3_ and Y_3_Al_5_O_12_ (YAG) as dosimeters for beta particles as noted on the bars, MgB_4_O_7_:Dy, (Ba_0.88_Sr_0.12_SO_4_)_99.8%_:Eu_0.2%_ (B88n), BaSO_4_:Eu, and LiF:Mg,Cu,P are for gamma ray dosimetry as noted, while CaSO_4_:Dy and BaSO_4_:Eu are HCP dosimeters.

Several promising nanomaterial TL techniques have been investigated to better understand their response to wider range of radiation beams. It is well known that traditional TL materials are mostly insulators or semiconductors on which irradiation with various doses of ionizing radiation, e.g., gamma rays, electrons, alpha particles, and heavy charged particles (HCPs), causes cold light emission when heated. TL materials development is widely utilized in the field of ionizing radiation dosimetry today. However, these materials have largely unchanged since the discoveries of TL techniques, and they are mostly used in low ionizing radiation dosimetry.^31,32^ Most TL materials, on the other hand, are not able to detect heavy charged particles (HCPs) accurately. The saturation effect is the most serious issue, and it occurs at low fluences, as well as the significant changes in the shape of the glow curves as fluence increases, which is unsuitable for dosimetry. In response, many researchers recently created nanocrystalline forms of sensitive phosphors and investigated their TL response to various heavy charged particles.^33–37^ These nanomaterials phosphors are useful for estimating the doses of HCP because of their linear response over a wide range of exposures, low fading, and insensitivity to heating treatments. Figure 1 demonstrates the dramatic increase in the upper detectable limit of dose for different nanomaterial-based TL dosimeters in comparison to their bulk structure. Some of these nanomaterials based on different cations, such as fluorides and sulfates, are discussed in this review from the viewpoint of dosimetry.

#### 1. Nano-fluorides as TL dosimeters

Lithium fluoride (LiF) is a radiation dosimetry phosphor with a high sensitivity in different types of radiation photons, electrons, neutrons, protons, as well as ions. When compared to photons, HCPs have a higher proportional biological efficacy and exhibit a stronger linear energy transfer (LET) with a sharper Bragg peak. Therefore, the dose of these heavy charged ions needs to be precisely determined spatially. For that reason, Salah *et al.* prepared lithium fluoride nanocubes doped with Eu and studied its TL response for 85 MeV C^6+^ ion beam. This nanomaterial was subjected to HCP radiation in pellet form at fluences ranging from 10^9^–10^13^ ions/cm^2^. The acquired data indicated a distinct TL glow peak at roughly 320 C, which was not caused by gamma rays. This glow peak had a linear response in the 10^9^–10^12^ ions/cm^2^ range, which corresponds to equivalent absorbed doses of 0.273–273 kGy. Using Monte Carlo (MC) simulation, the absorbed doses, penetration depths, and primary energy loss were computed using the TRIM code. Additionally, the stopping power and supralinearity factor of this nanomaterial were investigated. The glow curve structure variation of LiF:Eu nanocubes that is caused by changing the irradiation type might be useful for mixed field dosimetry. Furthermore, its wide linear response and low fading are central to the reason that this nanomaterial could be a promising candidate for carbon ion irradiation dosimetry.^33^

Another class of fluoride nanocomposites are calcium fluoride nanoparticles, which can have various dopants (Dy, Mn, Cu, and Tm).^45–48^ Slope of the dose response curve, on log-log graphs, for these TL dosimeters range from 0.81 to 1.33, and the rest of the features are summarized in Table II. Although CaF_2_:Cu nanoparticles have a lower TL sensitivity than GR-200 phosphor, they are more sensitive than the well-known TL dosimeter; LiF:Mg,Ti (TLD-100) chips. Moreover, it was reported that the dose response behavior and the linearity range of CaF_2_:Dy were significantly affected by the variation of the particle size, showing that the particle size of 65 nm increased the upper limit of detection to 12 kGy, from the previous 1 kGy at the 140 nm grain size.^49^ These data suggest that it could be a good dosimeter for high-dose applications when considering the proper TL dosimetry features, particularly the linearity over a large range of dose responses, and up to very high absorbed total dose.

**TABLE II.** TL-dosimetric features of the most recent prepared nanoparticle fluorides and sulfates. Slopes of these dosimeters were calculated from their log–log graph response.

Nanomaterial	Radiation	Linearity range	Slope	Fading over 30 days	Energy dependence	References
LiF:Eu	γ–rays	Strong sublinear: 0.01-30 kGy	0.24	7%	Slight	58
	C-ions	Sublinear: 0.273– 273 kGy ^a^	0.52	4%	Slight	33
LiF:Sm,Dy,Eu	γ–rays	Sublinear: 1-30 kGy	∼0.68	11%	Slight	59
LiNaSO_4_: Eu	γ–rays	Linear: 0.5 - 100 kGy	∼0.98	28%	Slight	60
CaF_2_:Dy	γ–rays	Supralinear: 3- 1000 Gy	1.33	∼5% for whole GC ^b^	Moderate	45
CaF_2_:Mn	γ–rays	Linear: 0.1 up to 3x10^4^ Gy	0.97	∼8% for peak 2	Moderate	46
				∼3% for peak 3		
CaF_2_:Cu	γ–rays	Linear: 1- 1000 Gy	1.04	∼10%	Moderate	47
CaF_2_:Tm	γ–rays	Nearly linear: 1- 1000 Gy	0.81	13%	Moderate	48
SrF_2_	γ–rays	Linear: 1- 1000 Gy	∼0.95	∼4%	Moderate	61
K_2_Ca_2_(SO_4_)_3_:Cu	γ–rays	Nearly linear: 0.01 to 300 Gy Sublinear: 0.3 to 1 kGy	0.760.65	N/A	Moderate	50
K_3_Na(SO_4_)_2_:Eu	γ–rays	Strong sublinear: 0.1 to 1 Gy Sublinear: 1 Gy to 1 kGy Strong sublinear: 1 to 40 kGy	∼0.4 ∼0.6 ∼0.4	∼6%	Moderate	62
MgSO_4_:Dy	γ–rays	Linear: 1 Gy – 10 kGy	1.08	∼67%	Moderate	63
MgSO_4_:Cu	γ–rays	Nearly linear: 1 Gy – 10 kGy	0.8	∼37%	Moderate	63
CaSO_4_:Eu	γ–rays	Nearly linear: 10 Gy – 10 kGy	0.8	∼12%	Moderate	64
CaSO_4_:Tb	γ–rays	Linear: 10 Gy – 5 kGy	0.9	∼6%	Moderate	64
CaSO_4_:Dy	C-ions	Sublinear: 0.3 kGy– 3 MGy ^c^	0.5	N/A	Moderate	36
	γ–rays	Supralinear: 1 Gy– 5 kGy	∼1.13	∼5%	Moderate	65
B88n	γ–rays	Linear: 0.2 mGy–0.6 kGy	1.02	Negligible	Strong	44,57
		Strong sublinear: 0.6-40 kGy ^d^	0.33			
						55
	Protons ^e^	Linear: 0.1 Gy – 2 Gy	1.01	Negligible	Strong	66
BaSO_4_:Eu	γ–rays	Linear: 0.1 – 1000 Gy	∼1	∼5%	Strong	67
		Sublinear: 1000-6400 Gy	∼0.74			
	Protons ^f^	Linear: 0.1 – 300 Gy	∼1.05	∼4%	Strong	

^a^ Equivalent dose of 10^9^–10^12^ ions/cm^2^ from 85 MeV C^6+^.

^b^ Negligible for GC with thermal cleaning of a low temperature peak.

^c^ Equivalent dose of 10^9^–10^13^ ions/cm^2^ from 75 MeV C^6+^.

^d^ These properties are for deconvoluted peak no. 5.

^e^ Protons with energy 35 MeV.

^f^ Protons with energy 150 MeV.

#### 2. Nano-sulfates as TL dosimeters

Nano-sulfate-cation compounds were generally synthesized by chemical co-precipitation method. Using this method, Mandlik *et al.* synthesized nanocrystalline K_2_Ca_2_(SO_4_)_3_:Cu phosphor, which was then annealed at a range of temperatures (400–900 °C) for 2 h. Nanorods of K_2_Ca_2_(SO_4_)_3_:Cu with diameter of 20 nm and a length of 200 nm were observed in the TEM images. The TL glow curve of this nanophosphor appeared at a maximum peak of roughly 175 °C and two peaks with low intensity near 85 °C and 305 °C. The OSL sensitivity of all three TL peaks was confirmed. Annealing at 700 °C was found to lead to the most sensitive approach, with a TL response that was linear from 0.01 to 300 Gy, and saturating gamma doses above 1 kGy as demonstrated in Table II. In the dose range of 0.01 Gy to 1 kGy, the OSL response was linear, before saturating at the highest doses.^50^

CaSO_4_ doped with Dysprosium with a particle size of roughly 30 nm is another nanophosphor that was prepared chemically by the co-precipitation method and investigated its dosimetric properties in an HCP beam. A 75 MeV C^6+^ ion beam with a fluence range of 10^9^–10^13^ ions cm^−2^ was used to irradiate pellet samples of these. The irradiated nanostructure had outstanding dosimetric properties, with a simple glow curve shape and linearity range of TL response that was wider than the corresponding microcrystalline sample as shown in Fig. 1. These findings suggest that the nano-CaSO_4_:Dy could be effective for detection of high carbon ion doses utilized in radiation therapy.^36^

A fascinating set of observations were obtained from the comparative study between CaSO_4_:Dy, K_2_Ca_2_(SO_4_)_3_:Eu, Ba_0.97_Ca_0.03_SO_4_:Eu, and LiF:Mg,Cu,P nanophosphors by Salah for HCP dosimetry.^37^ The nanomaterial phosphors are useful for estimating the doses of HCP because of their linear response over a wide range of exposures, low fading, and insensitivity to heating treatments. The findings described earlier clearly reveal that nanoscale TLD materials are well suited for ion beam dosimetry, as they are very sensitive to HCP over a wide exposure range and exhibit stable glow curves, especially for sulfate nanomaterials.^36,41,51^ The structures of the glow curves, as well as the positions of the peaks, did not change substantially. Their sensitivity was similarly equivalent to that induced by gamma irradiation. LiF:Mg,Cu,P nanorods had an entirely different behavior than sulfate nanomaterials.^52^ While this material was irradiated by gamma rays, it was discovered that the lower temperature peak at roughly 410 K responded linearly to γ-ray exposures, whereas the higher-temperature peak around 570 K disappeared. While bombarding this material with Li ions, the low-temperature peak disappeared and while the higher-temperature peak grew. Because the material has a Z_eff_ like that of tissues, and is frequently employed as a dosimeter in radiotherapy, this characteristic could be very advantageous. As a result, it works well with both lightly ionizing radiation like gamma rays and high LET HCP. It can be also used in space applications, where a variety of ionizing radiation is present. Sulfate materials, which have promised TL properties and a very linear TL response curve, especially CaSO_4_:Dy nanocubes, which could be employed as a standard TL dosimeter for carbon ion irradiation. Although the reaction of various nanostructures to ion beams varies, particularly in the case of sulfate nanomaterials and LiF:Mg,Cu,P (TL-100H), they all respond linearly across a wide range of exposures. This means that the number of traps formed within the host of these nanomaterials is greater than the number generated within their microcrystalline counterparts. The effect of heavy ions on the traps of these nanomaterials is the only distinction in this situation. This impact is more apparent in low-density host materials, such as LiF, than in sulfate materials. The traps inside this host were directly affected by the effect of HCP, since LiF is a simple two atom structure. When microcrystalline materials are irradiated by HCP, they exhibit another typical trend. The low-temperature peaks grew significantly faster than the higher-temperature peaks as the doses were increased as in CaSO_4_:Dy and K_2_Ca_2_(SO_4_)_3_:Eu microcrystalline.^36,53^ In this situation, the influence of these ions may modify the surface traps immediately, but the deep traps have less interaction. Owing to small grain size of nanomaterials, both the surface and deep traps have been altered. Furthermore, even though the deep traps produced inside the hosts of microcrystalline materials were slightly altered, they would still be unable to contribute to TL emissions. The generated photons by the deep traps may be absorbed via the microphase itself, preventing them from reaching the TL detector. As a result, saturation effects can dominate in microcrystalline materials, whereas these are less in nanomaterials. The small size of nanomaterial particles introduces a higher chance of being exposed and contributing to TL emission. Both the size of the heavy charged particles and their energy are important parameters that influence the TLD response range. When compared to nanomaterials irradiated by heavier ions, such as carbon and oxygen, lighter ions like lithium have less effect on the target material, hence the beginning of TL saturation occurs at greater fluences. The overlapping of tracks is greater in the case of these larger ions. These effects were found when Li^3+^, C^6+^, and O^7+^ ions had been used to irradiate K_2_Ca_2_(SO_4_)_3_:Eu and Ba_0.97_Ca_0.03_SO_4_:Eu nanoparticles.^37^

Nano (Ba_0.88_Sr_0.12_SO_4_)_99.8%_:Eu_0.2%_ (B88n) is a further sensitive TL nanophosphor prepared by Aboelezz *et al.* using a chemical co-precipitation approach and compared to its microstructure and TL-700.^44^ In the low dose range, the sensitivity results implied that nano- and microphases for B88 were around 50 times higher than TL-700. The gamma irradiated nanophosphor had a linear TL response over a range (1 mGy–2 kGy), whereas the irradiated microphosphor had a linear response over a dose ranging from 1 mGy to 750 Gy, after which it began to saturate. B88n samples had no fading issues, while microsamples faded rapidly. The energy dependence study revealed that B88 is energy dependent over all photon energies, except 0.8–4 MeV, due to its large effective atomic number, Z_eff_ = 43.5. For nano-B88, the lower detection limit (DL) for the irradiation regime was found to be 135 *μ*Gy. Because of the sensitivity of most TL dosimeters to UV, B88n should be kept in a dark area away from UV-light sources. The dosimetric features of B88n, together with low fading and good reusability, make it appropriate for radiation dosimetry applications after addressing the energy dependency problem. The combined uncertainty of TL measurements was ±5%, where it was found that the sample preparation and photomultiplier tube (PMT) noise components have the impact on this uncertainty budget.^44^

The effect of strontium addition on the thermoluminescence features of the (Ba_1−x_Sr_x_SO_4_)_99.8%_:Eu_0.2%_ nanophosphor series bombarded with gamma rays was examined.^54^ Samples with x = 0.12, B88n, and effective atomic number (Z_eff_ = 43.5) had the deepest trap centers and the best TL sensitivity. The complex B88n glow curve sub-peaks were examined experimentally and theoretically by computerized glow curve deconvolution (CGCD), showing that it approached first-order kinetics (FOK). The glow curve (GC) was composed of five overlapping peaks with somewhat different T_m_ positions when these approaches were compared. The influence of various heating rates of TL readout revealed that it followed FOK. A new attempt at the CGCD for nano-B88 was made using general order kinetics (GOK) analysis, by analyzing the five peaks at maximum temperature from 448 to 597 K, and activation energies from 1.05 to 1.74 eV.^44^ Because the GOK equation is based on an empirical formula, Hassan *et al.* studied the effect of gamma absorbed dose on R parameter instead GOK, for all the deconvoluted peaks.^55^ The physical parameter R was defined by the trapping-recombination ratio, $R=A_{n}/A_{m}$, where $A_{n}$ and $A_{m}$ are the trapping and recombination probability coefficients, respectively. The behavior of the kinetic order *b* and the trapping-recombination parameter R was theoretically explored.^56^ It was defined that the glow-peak when R ≪1, tended to the FOK model, whereas for R →1, the glow-peak shifted from FOK to second-order kinetics (SOK). They found that the geometrical properties of all deconvoluted peaks, except peak 5, were nearly constant over the dose range from 0.4 to ∼63 kGy. A linear response of peak 1 was found up to 4.5 kGy, making it a dosimetrically useful peak, while peak 5 might be used for the dose assessment with a sublinear response for gamma dose range (0.6–40 kGy). On the other hand, the area of the entire glow curve could be employed in dose estimation for gamma doses up to 2 kGy. Considering all of these features, B88n appears as an excellent potential dosimeter for personal, medical and high radiation levels.

Aboelezz and Elashmawy studied the signal stability and deconvolution of the B88n glow curve at low doses. There was no substantial change due to dose fractionation, and varying exposure rates, but still showed a linear response. The uncertainty percentage due to dose rate variation was around 0.44%, indicating that dose rate variation had a negligible influence. It is worth noting that along four decades of magnitude of gamma radiation dose rate the B88n (u_c_% = 1.1%) exhibited less dependence than the TLD-100 (u_c_%= 3.7%). There was also very little fading of this material. CGCD revealed the presence of a new peak at high temperature, approximately 633 K, that saturated at extremely low doses, less than 0.2 mGy, and faded away at greater doses. Further interesting properties of B88n, included independence on dose rates and dose fractionation, indicating it could be an excellent choice for a variety of radiation dosimetry applications.^57^ In summary, the strength of nano-fluorides and nano-sulfates in TL is that they can offer a wider linearity range of dose response as well as longer stability of the glow curve intensity when compared to their microcounterparts.

### B. Optically stimulated luminescence dosimetry

Optically stimulated luminescence (OSL) is one of the most important measurement tools of radiation dosimetry, especially in radiotherapy and protection fields. Here, nano-sulfate and aluminate families for OSL dosimetry of ionizing radiation are examined, in addition the improvement of OSL sensitivity through doping of plasmonic nanoparticles.

#### 1. Nano-aluminates for OSL dosimetry

A sensitive and accurate OSL dosimeter was made from the microstructure of aluminum oxide doped with carbon (Al_2_O_3_:C), by Akselrod and McKeever.^68^ The special characteristics of Al_2_O_3_:C include high ionizing radiation sensitivity, which has a low detection limit of dose. In addition, long luminescence lifetime of the recombination center leads to rapid pulsed-OSL measurement and slow OSL decay allowing for multiple dose determinations.^69^ The development of the commercial Luxel system was prompted by the well-established growth and manufacturing of Al_2_O_3_:C. During crystal development of alumina (α-Al_2_O_3_), the doping of carbon in a reducing environment increases oxygen vacancies. This lead to produce of F^+^ (one electron trapped) and F (two electrons trapped) centers, at an oxygen vacancy, respectively, which act as Al_2_O_3_ recombination centers.^70^ Later, many groups used various ways to fabricate nanostructured alumina samples and examined the ensuing luminescence characteristics. Kortov *et al.* used the gas phase approach to prepare aluminum oxide nanopowders and investigated their photoluminescence (PL) and cathodoluminescence.^71^ The materials possessed many of the same luminescence properties as anion-deficient Al_2_O_3_, although the existence of oxygen-vacancy non-equilibrium phases caused new luminescence bands to develop. The PL and TL of nanostructured Al_2_O_3_ generated by a combustion synthesis process were also investigated, and at least one of the TL peaks was determined to be appropriate for radiation dosimetry.^72^ These research studies showed variations between nanophosphor and bulk materials; however, the discrepancies could not be attributed to the small dimensions of nanophosphors. The quantum confinement effects observed in semiconductors^73^ do not apply to nanocrystalline insulators in general. Reduced dimensionality in nanocrystalline insulators has been linked to the non-uniform broadening of emission lines, different populations of the excited state,^74^ and variations in luminescence lifetimes,^75^ but these phenomena should have no direct impact on TL or OSL dosimetry. Nevertheless, the enhanced surface/volume ratio and decreased scattering of small particles could have a major impact on dosimetric nanophosphor performance. Therefore, Blair *et al.* used solution combustion synthesis (SCS) technology to make Al_2_O_3_ nanophosphor powders, which is capable of high output production of nanostructured materials.^76^ All nanophosphor Al_2_O_3_ samples that were prepared using different fuels have a greatly reduced luminescence intensity by a factor of more than 100, in comparison to bulk Al_2_O_3_:C.

The existence of oxygen vacancies, in the material because of its growth conditions is thought to be the main reason for the intense OSL response of bulk Al_2_O_3_:C.^77^ Thus, the reduced TL and OSL intensity of the nano-Al_2_O_3_ may be attributed to fewer oxygen vacancies in the crystal lattice, which is produced by the solution combustion synthesis SCS process in other oxides.^78^ Although the Al_2_O_3_ nanophosphors show potential as OSL radiation dosimeters, their applicability is currently limited due to their lower luminescence properties. This limitation is in nanophosphor luminescence and non-monotonic dose response curves as seen in some samples. In addition, there is SOK behavior in their TL glow curves.^76^ However, this comparison is not entirely complete because the prepared alumina NP were not compared comparably to their bulk counterpart, at the chemical formula; it was just compared to the most used reference OSL dosimeter. Furthermore, the optimum conditions of nanomaterial synthesis, such as dopants, concentrations, annealing procedures, and fuels, were not studied for optimization yet.

#### 2. Nano-sulfates for OSL dosimetry

Patle *et al.* synthesized a highly sensitive nanophosphor of SrSO_4_:Eu by annealing at 1000 °C, creating optically stimulated luminescent phosphor. The fabricated nanophosphor has excellent OSL properties, showing a sensitivity of 1.26 times that of a commercially available Al_2_O_3_:C phosphor using the area integration approach. While the integral area of a bulk SrSO_4_ is 24% of the integral area of a commercially available Al_2_O_3_:C. This indicates that the sensitivity of nano-SrSO_4_:Eu was five times greater than that of its bulk counterpart. After the OSL readout, the SrSO_4_:Eu sample revealed a single TL glow peak around 230 °C, which was determined to be reduced by 47%. This sample had a sublinear dose response up to about 100 mGy and then tended to become strongly sublinear, indicating that it is extraordinarily sensitive and, thus, appropriate for detecting very low dose levels.^79^ As it is known that the absorption efficiency of the laser by NP is higher than that of larger grain-sized material.^80^ Since the OSL reader is equipped with a blue LED laser source that is utilized for stimulation of very deep traps (VDTs),^81^ this led to liberating more charge carriers from these luminescence traps in the NP. This absorption efficiency factor could explain why OSL dosimeters based on nanomaterial are more sensitive than their microstructured counterparts.

B88n irradiated with different beam qualities in the dose range (0.07–2 Gy) was studied, and exhibited a linear relationship of dose response in both TL and OSL signals.^66^ A weakness of all barium compounds is the fact that they are not tissue-equivalent. The energy dependence factor (*F_E,Cs_*)^82^ was evaluated in terms of absorbed dose to water. For all beam qualities with the exception of the 15 MV beam, the measured *F_E,Cs_* of B88n using both TL and OSL tools coincided with the expanded uncertainty of the estimated *F_E,Cs_* with a coverage factor of 3. Furthermore, the standard error of the MC simulation of mean photon spectrum energy was 2%. Also, they found that TLD-100 and Al_2_O_3_:C, on the other hand, exhibited a considerable energy dependence as compared to nano-B88. Hence, correction coefficients for energy dependency were required for more accurate dosimetry measurements. Under the conditions studied, the estimated detection limits for the B88n/TL system were near 48 mGy for ^137^Cs and 180 mGy for 16 MeV electrons, whereas for the B88n/OSL system, they were 122 mGy for 6 MV photons and 153 mGy for 16 MV photons. However, by employing smaller dosages and increasing the number of data points, these OSL limitations could be decreased. These outstanding characteristics indicate that this dosimeter could be a superb option for TL and OSL dosimetry in radiation therapy. Comparisons between factors of the measured energy dependence, $F_{E,Cs}$, for B88n and, TL and OSL reference dosimeters from the literature are illustrated in Tables 5 and 6 in Ref. 66. Thus, nano-sulfates in the OSL dosimetry system appear advantageous for their lower detection limit, relatively stable intensity in their decay curve, and with a superior sensitivity to the same material in a microphase vs a nanophase.

#### 3. OSL dosimetry improvement using plasmonic nanoparticles

Many materials, including oxides (Al_2_O_3_, BeO, and MgAl_2_O_4_) and alkaline halides (NaCl, KCl, and KBr) have OSL signal. Ionizing radiation mostly induces trapped electrons; OSL emission occurs only by releasing the trapped electrons through sufficient light energy stimulation.^3,83,84^ Host doping and co-doping with additional elements (magnesium, carbon, boron, and rare earths) to enhance the number of trapping and/or luminescence centers, are traditional ways for improving the radiation sensitivity of OSL materials, as in Al_2_O_3_:C, NaCl:Eu^+2^, and KCl:Eu^+2.^^85^ In this context, Guidelli and co-workers discovered a novel way of increasing OSL intensity through the interaction of plasmonic nanoparticles with luminescent centers. The hypothesis is that the plasmon absorption band of these dosimeters results from the resonance of the collective oscillation of electrons in the conduction bands on the surfaces of NPs and the electric field of the incident light.^86^ Local electromagnetic field amplification may be obtained under this kind of resonance settings, which is utilized to improve luminescence processes, such as fluorescence and phosphorescence among others.^87^ This method is used for improving OSL material sensitivity, as an alternative to the usual doping method. They chose silver as a noble metal particle because the blue OSL stimulation band (460 nm) exactly matches the plasmon resonance band of silver nanoparticles (AgNPs), increasing the probability of an amplified local field around the AgNP. It was also critical to choose the right luminescent substance after choosing the optimum metal nanoparticle. Alkaline halides, among the OSL materials, are favorable because they are soluble materials and facilitate crystal growth at low temperatures around the silver nanoparticles. Guidelli *et al.* investigated plasmon enhanced NaCl^88^ as an OSL dosimeter,^89,90^ because of the overlap of plasmon resonance bands of silver nanoparticles and the F-center absorption bands of NaCl.^91^ Another attempt was made to improve the OSL dosimetric properties of microstructured CaSO_4_ doped with Terbium (Tb) by co-doping with AgNP and comparing it to that co-doped with micro Ag.^92^ In that work, the dosimetric features were enhanced by lowering the fading and increasing the sensitivity, leading to a decrease in the detection limit.

**FIG. 2.**
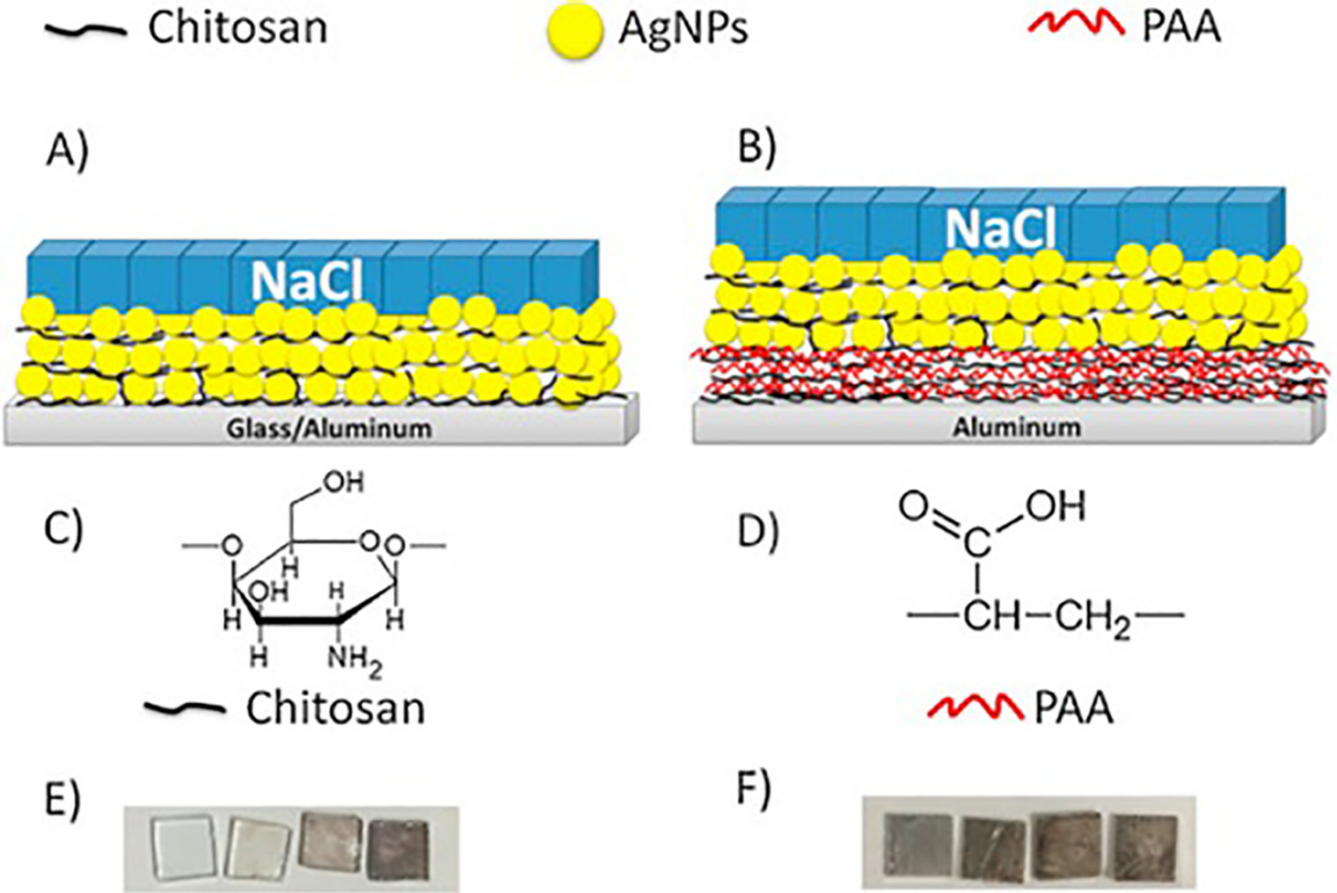
Sketch of NaCl doped with AgNPs detector system. (a) the film of chitosan doped with AgNP coated on glass and aluminum substrates then covered by NaCl on the top. (b) Chitosan/PAA covered by chitosan/AgNP films precipitated above aluminum substrates with top deposition of NaCl. (c) and (d) The chemical formula of chitosan and PAA. (E) and (F) The AgNP films deposited on glass and aluminum. From left to right, the number of precipitated layers from chitosan/AgNP increases. Reproduced with permission from Guidelli *et al.*, Sens. Actuators B **224**, 248 (2016). Copyright 2016 Elsevier.^87^

Additionally, the plasmonic AgNP films using the layer-by-layer (LbL) technique were prepared, by alternating nanoparticles and polyelectrolyte; chitosan and polyacrylic acid (PAA); in layers as shown in Fig. 2.^87^ It was found that as the AgNP layers increased the OSL intensity, allowing for increased detection sensitivity proportionately. A significant OSL increase was seen for films placed over glass substrates, due to the plasmonic connection of the x-ray generated luminescent centers and the plasmons at the surface of nanoparticle film. Additionally, AgNP films produced on aluminum substrates resulted in a negligible increase in OSL. Controlling the number of implemented AgNP layers on the glass substrate, or the distance between the nanoparticle layer and the aluminum substrates, could improve detection sensitivity. These findings suggest that silver nanoparticle films could be used to build more sensitive and smaller detectors for medical applications of ionizing radiation. The main characteristics of the most recently demonstrated nanoaluminates and sulfates as OSL dosimeters are summarized in Table III.

**TABLE III.** The main characteristics of the most recent prepared nanoaluminates and sulfates as OSL dosimeters. Slopes of these dosimeters were calculated from their log–log graph.

Nanomaterial	Radiation beam	Linearity range	Slope	Fading	Energy dependence	References
Al_2_O_3_	β-particle^b^	Linear: 0.8–50 Gy^d^	1.02	NA	Slight	76
K_2_Ca_2_(SO_4_)_3_:Cu	γ-rays	Linear: 0.01–1000 Gy	0.9	Negligible	Moderate	50
K_2_Ca_2_(SO4)_3_:Eu	γ-rays	Linear: 0.01–1000 Gy	1.02	NA	Moderate	93
CaSO_4_:Tb,AgNP^a^	β-particle^b^	Linear: 0.17–10 Gy	1	NA	Moderate	92
SrSO_4_:Eu	β-particle^b^	Nearly linear: 40–100 mGy	0.76	Negligible (non-destructive readout)	Strong	79
		Strong sublinear: 0.1–1 Gy	0.39		
B88n	γ-rays	Linear: 0.01–2 Gy	0.98	23% after 4 days then stabilized	Strong	66
	Protons^c^	Linear: 0.1–2 Gy	1.05	NA	Strong	
BaSO_4_:Eu	β-particle^b^	Nearly linear: 0.1–1 Gy	∼0.83	5% after 4 days then stabilized	Strong	94

^a^ Concentration of both Terbium (Tb) and AgNP was 0.1 mol. %. ^b^ β-source was ^90^Sr/^90^Y.

^c^ Protons at 35 MeV.

^d^ synthesized with urea as a fuel.

### C. Scintillators

**FIG. 3.**
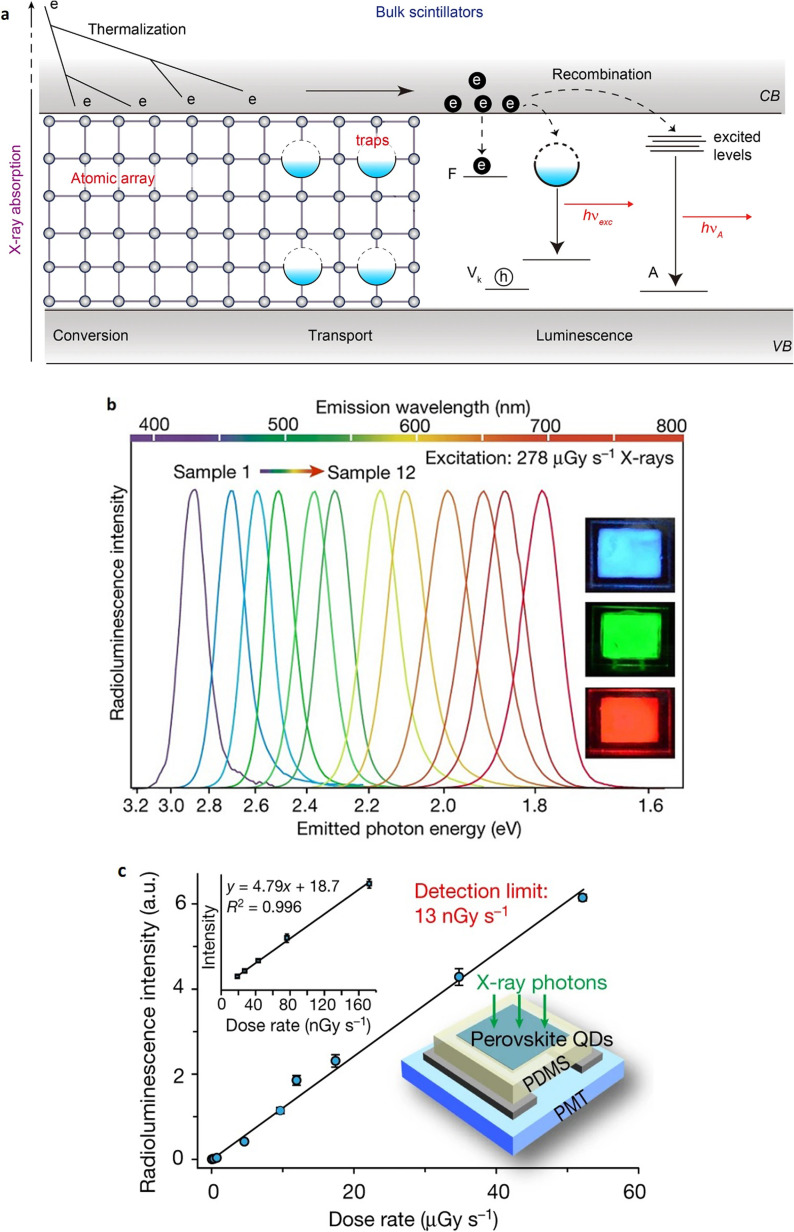
Scintillation hypothesis in bulk inorganic materials (a). V_k_, self-trapped hole; F, Farbe center; *ν*_exc_, frequency of photon during excitons luminescence; *ν*_A_, photon frequency in activator (A) luminescence, VB, valence band; and CB, conduction band. Luminescence spectra of the perovskite QDs at a voltage of 50 kV of x-ray irradiation with a 278 *μ*Gy/s dose rate (b). Twelve separate spectra for materials composed of (1) CsPbCl_3_, (2) CsPbCl_2_Br, (3) CsPbCl_1.5_Br_1.5_, (4) CsPbClBr_2_, (5) CsPbCl_2.5_Br_0.5_, (6) CsPbBr_3_, (7) CsPbBr_2_I, (8) CsPbBr_1.8_I_1.2_, (9) CsPbBr_1.5_I_1.5_, (10) CsPbBr_1.2_I_1.8_, (11) CsPbBrI_2_, and (12) CsPbI_3_. Thin-film samples 3 (blue), 6 (green), and 9 (red) are shown in the insets, upon x-ray exposure. (c) Measurements of radioluminescence for a CsPbBr_3_-based scintillator in relation to dose rate. Radioluminescence profiles recorded at low dose rates are displayed in the left inset. The slope of the fitting line was used to determine the detection limit, which was 13 nGy s^−1^, at a signal-to-noise ratio (SNR) of 3. A schematic of the x-ray photodetector is seen in the right inset, containing three layers including a thin coating of CsPbBr_3_ nanocrystals (≈120 m thick), a polydimethylsiloxane (PDMS) layer, and a photomultiplier tube (PMT). Reproduced with permission from Chen *et al.*, Nature **561**, 88 (2018). Copyright 2018 Springer Nature.^104^

The growing demand on radiation detection materials in multiple applications has resulted in substantial research into both organic and inorganic scintillators.^95–99^ The absorption capability of most organic and inorganic scintillators, discussed in this review, are crucial for high-energy (keV) x rays and conversion of the absorbed energy to visible photons with low energy for applications, such as radiation detection, x-ray astronomy, security inspection, and medical radiography.^100^ There are to date far too few direct conversion materials that can economically substitute nanoscintillators for general x-ray imaging with integrated detectors, with the exception of selenium (Se) detectors for mammography.^101^ The incident x-ray photons on common bulk inorganic scintillator can interact with high Z atoms (e.g., lead, thallium or cerium) to generate a large number of primary electrons through the photoelectric effect.^102^ These charge carriers are thermalized rapidly and create low-energy excitons, which are then transferred to defect sites or traps for radiative luminescence as shown in Fig. 3(a). However, the fabrication of the conventional scintillators is through high-temperature crystallization, and their radioluminescence (RL) is hard to adjust over the visible light spectrum. The unique photophysical properties of nanomaterials include the control of electron–hole pairs and the effective generation of multiple excitons, i.e., the production of several electron–hole pairs by a single photon. The electron–hole pairs in bulk scintillators lose their energy as phonons and are positioned at the edge of the luminescence trap. Whereas the excited electrons can transfer their energy through coulomb interaction to create new electron–hole pair rather than emitting phonons. As a result, in contrast to a bulk scintillator, a scintillator based on nanomaterial can control the emission wavelength and increase efficiency depending on the type and size of the employed nanomaterial. Another benefit of using nanomaterials with high Z_eff_ is the ability of the enhancement of the reaction rate with photons.^103^

In the last few years, experimental results from a variety of inorganic and organic perovskite nanocrystals (NCs) and other nanoscintillators, as well as their x-ray irradiation responses, have been reviewed comprehensively and in more details.^101,103–105^ At visible wavelengths, these nanocrystal scintillators have robust x-ray absorption and intense RL. These perovskite nanoparticles, unlike bulk inorganic scintillators, may be prepared at a relatively low temperature and can induce controllable x-ray emissions across the visible spectrum by modifying the anionic motif of the colloidal reactants during the production. These characteristics are summarized in Table IV and enable manufacturing of adaptable and sensitive detectors which work at very low dose rates, with a lower detection limit of 13 nGy/s [Fig. 3(c)]. This rate was approximately 420 times lower than usual medical imaging doses (5.5 *μ*Gy s^−1^).^106^ The radiation induced luminescence of CsPbBr_3_ QDs has been reported to be 7600 times stronger than that of bulk crystals. These scintillators are color-tunable perovskite nanocrystal, which may be provided as an easy-to-use visualization material for x-ray radiography because the associated image can be captured immediately with standard digital cameras. Also, Heo *et al.* synthesized a new less expensive CsPbBr_3_ NCs-based scintillator as an indirect-type x-ray detector with high stability and resolution and a relatively low response time.^107^ Owing to the high photoluminescence quantum yield (PLQY) of the CsPbBr_3_ NCs scintillator compared with the conventional gadolinium oxysulfide (GOS) scintillator, it exhibited a higher ratio of light output power. It was found that the response time of the NCs sample was about 200 ns, which is more than fivefold faster than the GOS sample. The spatial resolution of CsPbBr_3_ NCs has improved in comparison to GOS, from 6.3 to 12.5–8.9 lp mm^−1^, respectively. This loss in resolution was attributed to the extreme low crystal size of 9 ± 1.5 nm, which leads to reduced light scattering and consequently deteriorated spatial resolution in x-ray images. In addition, the CsPbBr_3_ NCs scintillator shows a comparable stability of the analog-to-digital converter (ADC) output of the detector with respect to the x-ray dose of only 4% over a wide dose rate range (0–40 Gy_air_ s^−1^). It was also reported that CsPbBr3 NCs scintillators have exceptional radiation hardness even at severe levels of radiation as high as 1 MGy, indicating their suitability for detection in harsh radiation environments.^108^ In light of this, the large area of CsPbBr3 NCs scintillator could be of value for future industrial readout electronics, diagnostic devices, or nuclear reactors.

**TABLE IV.** The main characteristics of the most recent prepared nano scintillators for radiation detection.

Compound		Detection particle		Decay time (ns)		Scintillator (Sc) type		Light yield (photons. MeV^−1^)		DL (nGy/s)		Energy dependence		References	
MAPbBr_3_-toluene		X- and gamma ray		59.8		Inorganic-organic based on liquid SC		NA		300		Slight		113	
CsPbBr_3_-dye-PMMA		X-ray		3.4^d^		Inorganic-organic		∼9000		NA		moderate		110	
		α-particle		NA		Inorganic-organic		∼1000							
CsPbBr_3_:Lu^3+^^a^		X-ray		27		Inorganic		NA		50		Strong		114	
KLGF–GC^b^		X- and gamma ray		53.6		Inorganic		853		NA		Strong		115	
Nano-FOD		X-ray		2.1 × 10^6^		Inorganic		NA		NA		Strong		112	
CsPbX_3_ QDs^c^		X-ray		44.6		Inorganic		∼21 000–29 000		13		Very strong		99, 104	
CsPbBr_3_ NCs		X-ray		200		Inorganic		∼66 000		NA		Very strong		107	
Cs_3_Cu_2_I_5_		X-ray		38.9		Inorganic		∼80 000		NA		Very strong		98	

^a^ Embedded within an inorganic transparent amorphous medium.

^b^ The chemical composition is 70SiO_2_–15KF–5LaF_3_–10GdF_3_–0.5CeF_3_.

^c^ (X = Cl, Br or I).

^d^ the lifetime of fast component of sensitized luminescence.

A further series of perovskite nanophase (CsPbX_3_, with X = Cl, Br, or I) were prepared via a hot-injection solution method,^109^ with a mean size of 9.6 nm. Upon x-ray beam irradiation, the prepared perovskite quantum dots (QDs) yield uniquely narrow and color-tunable emissions (1.7–3 eV), realizing multi-color, high-efficiency x-ray scintillators as appeared in Fig. 3(b). In contrast to the conventional bulk scintillators like CsI:Tl, YAlO_3_:Ce, PbWO_4_, and Bi_4_Ge_3_O_12_ (BGO), the emitted radioluminescence spectrum was wide, with a large full width at half maximum (FWHM) and almost unchangeable. Perovskite nanocrystal scintillators have a number of advantages, contrary to conventional CsI:Tl scintillators, including low toxicity, low-temperature solution synthesis, very fast scintillation response, and high emission quantum output.

Owing to the composition of low-density plastic scintillators from low Z_eff_ materials, they can be fabricated in a variety of shapes and sizes and used for beta ray detection, which makes them widely used in whole-body counters and radioactive monitoring systems. Nevertheless, their nuclear species resolution is poor due to the nature of radiological detectors. Inorganic scintillators or semiconductor detectors are, thus, implemented for precise radiological monitoring. Inorganic scintillators are preferably used for gamma-ray spectra because they have reasonable scintillation efficiency rates and resolutions but manufacturing them in large quantities is challenging and expensive. Therefore, fabricating plastic scintillators with performance qualities comparable to those of an inorganic scintillator makes them advisable for nuclear medicine measurements as well as measurements at large-capacity deconstructed waste sites. New electrical, optical, and magnetic properties are produced when nanomaterials are added to plastic-based materials, according to recent studies. Therefore, it is anticipated that high performance plastic detectors can be created by increasing the luminescence efficiency if nanomaterials are used.

**FIG. 4.**
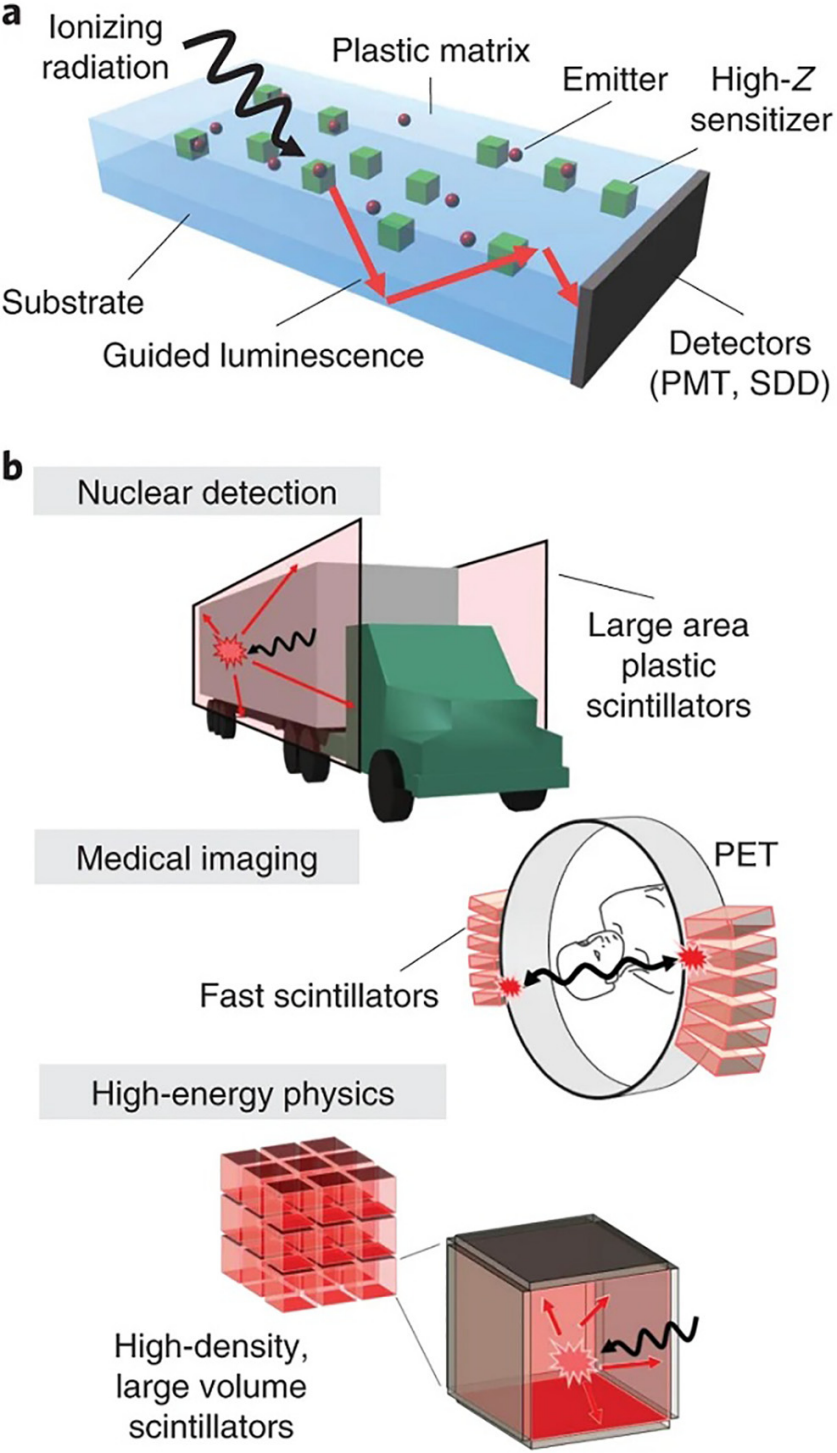
Concept and applications of plastic scintillators based on perovskite. (a) and (b) Schematic diagram of plastic scintillator enhanced with high-Z (a), the luminescence resulted from interacting ionizing radiation with the scintillator reflected totally by waveguide edges and then detected by a photomultiplier tube (PMT) or silicon drift detector (SDD). Applications in nuclear materials detection using large area plastic scintillators, fast timing medical imaging in positron emission tomography (PET), and physics of high-energy with high event rates (b). Reproduced with permission from Gandini *et al.*, Nat. Nanotechnol. **15**, 462 (2020). Copyright 2020 Springer Nature.^110^

As mentioned above, the establishment of scintillator devices that combine efficient scintillation, high probability of ionizing radiation interaction, rapid emission lifetime, and attenuated reabsorption losses in large volume/high-density detectors in fields of medical diagnosis, nuclear control, and particle physics has proven to be a difficult task. Nevertheless, it was achieved using poly methyl methacrylate (PMMA) nanocomposites with CsPbBr_3_ perovskite nanocrystals embedded as catalysts for a low Z conjugated organic dye (scintillating material) with a significant Stokes shift and a short lifetime of the emission in red wavelength range.^110^ Figures 4(a) and 4(b) depict the fundamental concept of the fabricated device as well as potential applications for its use. Using both x rays and α-particle irradiation, complete sensitization of the selected dye (the perylene dyad 9,9′-bis[perylene-3,4-dicarboxylic-3,4-(N-(2,5-di-*tert*-butylphenyl)]) by the perovskite nanocrystals leads to stable RL with an efficiency comparable to BGO crystal and (Kuraray SCSF-3HF), which are commercially graded inorganic and plastic scintillators, respectively. This device has a quick emission lifetime of 3.4 ns, which is comparable to efficient lanthanide scintillators, as well as negligible reabsorption loss at extended optical distances, showing its capacity to detect photons and charged particles with minimal reabsorption losses.

There are still certain restrictions on the use of lead halide perovskite-based scintillators, such as instability and the use of a heavy metals (i.e., lead), even though these materials have demonstrated a greater PLQY than typical x-ray scintillators. Therefore, new family of lead free Cs_3_Cu_2_I_5_ nanocrystals (NCs) scintillator have been tested using a low-temperature solution method for high-performance x-ray CT imaging application.^98^ The PLQY of the Cs_3_Cu_2_I_5_ NCs obtained a high value of 59% because the usual crystal structure efficiently increased the PL by restricting exciton access to the photoactive sites. Additionally, the PL mechanism of exciton self-trapping might explain some remarkable features, such as the significant Stokes shift (145 nm) and comparatively long PL duration (1085 ns). A fast response to x-ray stimulation was also demonstrated by the Cs_3_Cu_2_I_5_ NCs, with a scintillation decay time of only 38.94 ns. RL light yield of the Cs_3_Cu_2_I_5_ NCs is four times more than that of the CsPbBr_3_ NCs (80 kV, 70 A). As a high-efficiency x-ray scintillator, Cs_3_Cu_2_I_5_ NCs are excellent for ultra-sensitive and versatile x-ray detection. As Cs_3_Cu_2_I_5_ is inexpensive and has great environmental stability, it can be used as a next-generation x-ray scintillator for commercial medical diagnosis and industrial nondestructive testing. In this work, the researcher succeeded in reconstructing 3D CT picture of a snail and obtaining a clear projection image of a chip.

The nanocrystal of [Y_1.9_O_3_:Eu_0.1_,Li_0.16_] (70 ± 20 nm) is another inorganic nanoscale scintillator that was developed and studied for its response to x-ray energies (40–220 kVp).^111^ This nanomaterial was fabricated as a pellet and attached to one end of the UV/vis optical fiber terminals to build a prototype nanoparticle-dissipated fiber-optic dosimeter (nano-FOD).^111,112^ Belley *et al.* demonstrated nano-FOD opens new options for *in vivo* dose assessments and online data acquisition in case x-ray microbeam irradiation. Moreover, it promotes a new tool to measure the continuous dose rate at a microbeam peak.^112^ The nano-FOD may be used to estimate the peak-to-valley dose rate (PVDR) of multi-collimated microbeams and detect any finite beams number in a parallel array. The average dose exposed from 160 kVp beam in the microbeam peak of x ray agreed with that of radiochromic film, revealing that the nano-FOD is an excellent candidate for real-time microbeam radiation therapy (MRT) dosimetry. This device contrasts sharply with the capabilities of radiochromic film dosimeters, which need equilibrium times of multiple hours between irradiation and the readout result.

Briefly, nanoscintillators have the potential properties outlined above that make them suitable for the following applications: enhanced ionizing radiation detection sensitivity, such as microscopic diagnostics of biological and medical objects.^97^ They could also have potential *in vivo* biomedical uses such as in x-ray-induced photodynamic therapy (XPDT) as translators from x ray to optical excitation in medicines, although this work is still in early pre-clinical testing.

### D. Electron spin resonance (ESR)

This kind of spectroscopy has acronyms, such as electron spin resonance (ESR), electron paramagnetic resonance (EPR), or electron magnetic resonance (EMR) spectroscopy,^116^ so both ESR and EPR abbreviations have been used in this review, but we note here that they generally refer to the same or similar spectroscopic dosimetry techniques. In this section, only nano-sulfate composites and plasmonic nanoparticles are discussed as they are used in the alanine reference dosimeter, showing their feasibility in radiation dosimetry using EPR readout methods.

#### 1. Nano-sulfate for EPR dosimetry

A comparative study in ionizing radiation dose response of electron paramagnetic resonance (EPR) between the microphase of BaSO_4_ and its nanostructure prepared by the co-precipitation method was performed.^117^ EPR signals of both prepared nano- and micro-BaSO_4_ samples were attributed to SO_4_^−^ (hole center) and SO_3_^−^ (electron center). The spectroscopic splitting factor (g) of dosimetric signals for the prepared nano- and micro-BaSO_4_ was at 2.0025 and 2.0027 (σ = 0.0006), respectively. Nano-barium sulfate sample displays a linear dose response in the 0.4 Gy–1 kGy range, with a combined uncertainty of 3.9%, showing nano-BaSO_4_ is 1.5 times more sensitive to than alanine. A thermal stability analysis was used to calculate the lifetime and activation energy of nano-BaSO_4_, which are 57 ± 11 ka and 0.73 ± 0.14 eV, respectively. In addition to the high sensitivity and insolubility in water of the nano-BaSO_4_ sample, there is almost no ESR intensity fading. However, Aboelezz *et al.* proposed this dosimeter, particularly in a humid environment, for radiation dosimetry applications.^117^

The EPR response of materials as a function of different radiation qualities is a critical challenge, hence nano-BaSO_4_ response against radiation quality (6 and 15 MV photons, 6 and 16 MeV electrons, 35 MeV protons, and ^137^Cs as a reference beam), dose range from 0.3 to 55 Gy, was explored.^118^ It was reported that despite no beam quality affected linearity of dose response and signal line shape, the slopes of all beams have been differed substantially. The energy dependence factor was estimated to be about 0.6 for protons, 0.70 for electrons, near to unity for 6 MV photons and 0.87 for 15 MV photons. A comparison of measured energy dependence factors for nano-BaSO_4_ and the EPR reference dosimeter (alanine) from the literature is shown in Table 4 of Ref. 118. According to the findings, nano-barium sulfate has a significant energy dependency, necessitating the use of energy dependence correction factors when employing this material for dosimetry. This research also showed that the calibration-based detection limit of nano-barium sulfate varied from 111 mGy (^137^Cs) to 517 mGy (6 MeV electrons). Therefore, the nano-sulfates/EPR system can add value to the dosimetry by providing (1) higher sensitivity than both the bulk and the reference EPR dosimeter (alanine), (2) a wider dynamic range than its bulk, (3) longer lifetime stability than the bulk, and (4) working in a humid environment.

#### 2. ESR dosimetry improvement using plasmonic nanoparticles

Enhancement studies of radiation dose caused by adding heavy nanoparticle atoms (i.e., having high Z and dense), like gold, to the target just before irradiation have been done primarily using cells in culture,^119,120^ and theoretically,^121,122^ and are reviewed here.^123^ A qualitative assessment of the dose enhancement (DE) level of irradiated alanine/gold and silver nanoparticles complex to a limited energy range of low x rays.^124^ Smith *et al.* quantified the DE of irradiated alanine/AuNP/wax composites in a range of 2–20 Gy from low and high energy x ray, electron, and proton beams and examined the generation of radical species caused by gold nanoparticles as well. In a wax pellet, DL-alanine was doped with 5 nm AuNPs (3% by weight), which is the optimum concentration as reported by Guidelli and Baffa,^124^ and applied as a uniform layer. For 80 kV x rays, it was found that the gold nanoparticles enhanced the dose by more than 60% at ≈5 Gy, while smaller dose enhancements for MV x-ray beams (up to 10%) were observed, at the same measurement conditions. The dose enhancement induced by charged particles was found to be low for 6 MeV electrons (about 5%) but less than that for 150 MeV protons. The proton results confirm the most recent MC simulation results, although the radiosensitization is substantially lower by about 20% than that shown in cell and animal model systems. This discrepancy is due to the fact that alanine only assesses free radical levels created by nanoparticle inclusion, not redox type radicals (e.g., reactive oxygen species) formed by aqueous media in cells. It may also be attributed to the fact that the distribution of enhanced dose caused by AuNP on the nanoscale is inhomogeneous, rather than variations in dose over the entire dosimeter. The small range of these inhomogeneities and the diversity in enhancement across cells show that sub-cellular localization is significant in determining nanogold radiosensitization.^125^ In conclusion, they suggested utilizing the alanine/AuNP composites as highly sensitive alanine dosimeters for use with lower-level radiotherapy.

Later, Geso and group estimated the dose enhancement yielded by AuNP/alanine composites using L-alanine instead DL-alanine.^126^ They investigated that DE raised with growing AuNP concentration in both kV and MV x rays, with 80 kV x rays having the lowest percentage of DE; 15% for the 1% AuNP/alanine composites, up to 55 percent for the 3% gold. However, proton irradiation (150 MeV) had no such effect on AuNP concentration and consistently caused DE levels of less than 5% for all AuNP concentrations tested.

Dose enhancements of another derivative of alanine, namely, 2-methyl-alanine (2MA), impregnated with AuNP was estimated^127^ as the factor improvement in slope for the same dose values. With 5 nm AuNP at concentration of 0.1% w/w embedded in 2MA was irradiated to doses (up to 5 Gy) from 250 kV orthovoltage and 6 MV Linac. Dose enhancement was observed for small doses (0.1–0.5 Gy), with a kV beam having stronger impact, whereas for doses between 0.5 and 5 Gy, it was stable and independent of dose for both beams, about 2.1 and 1.3 (σ = 33%) for kV and MV beams, respectively. They also found that dose enhancement factors (DEFs), given as the slope of dose response for AuNP/2MA, to the slope for undoped 2MA, was 1.62 ± 0.04 to 1.27 ± 0.03 for the same beams. It is worth noting that AuNPs did not interfere with the ESR equipment, including microwave stimulation, magnetic fields, and paramagnetic radicals. In the presence of AuNPs, 2MA was shown to be a viable paramagnetic material for radiation dosimetry, and ESR dosimetry to be a robust experimental technique for additional verifications of nanoparticle-mediated doses of biological relevance. In the end, gold nanoparticles exposed to kV or MV beams can produce large and observable DE in biologically like materials.

Recently, nanocomposites of alanine (Ala), 2-methylalanine (2MA), asparagine (Asn) and monosodium glutamate (MSG) with various mass % of AuNPs from 0.1 to 3% were compared for dosimetric applications.^128^ Additionally, the influence of intrinsic sensitivity of these materials and the absorbed dose (2 Gy–10 kGy) and on DEFs were investigated. The findings showed that the AuNP aggregated in the irradiated MSG and Asn molecules but did not in the Ala or 2MA nanocomposites. Higher DEFs were reported for materials with lower intrinsic sensitivity (Asn and MSG) and for lower doses of radiation, suggesting that the probability of radical recombination regulated the dosimetric response of the nanocomposite dosimeters. A large correlation between DEF and the dosimeter sensitivity, AuNP mass percent, and the delivered dose was observed. Higher DEFs were found for dosimeters with lower sensitivity (Asn and MSG) and lower radiation doses, implying that the dose response of nanocomposites is determined by the radical recombination probability. From the theoretical calculation of DEFs using “Physical Reference Data”—National Institute of Standards and Technology (NIST),^129^ it was found that only the theoretical DEF for the 1% AuNP mass percentage and 2 Gy dose agreed with experimental results. They attributed this deviation between the theoretical and experimental results to neglecting the self-absorption of absorbed dose by nanoparticles.^124^ The dosimetric features of the prepared nanomaterials and hybrid composites using the EPR technique are shown in Table V.

**TABLE V.** EPR-dosimetric features of the prepared nanomaterials and hybrid composites. Slopes of these dosimeters calculated from their log-log dose response graph.

Compound	Material type	Linearity range	Slope	Fading	Z_eff_	Energy dependence	References
2-methylalanine/ AuNP 0.1% w/w	Hybrid composites^b^	Linear: 0.1–5 Gy	0.9 and 0.93^d^	NA	9.3	Slight	127
DL-Alanine/AuNP 3% mass%	Hybrid composites^b^	Linear: 2–20 Gy	0.85–1^e^	NA	24	Moderate	130, 131
L-alanine/AuNP 1,2,3% w/w	Hybrid composites^b^	Sublinear/Linear: 2–20 Gy	0.73–0.95^f^	NA	16.8, 21, and 24^g^		126
BaSO_4_	Nanomaterial	Linear: 0.3–1000 Gy	1.04	Negligible	46.9	Strong	117
		Linear: 0.3–55 Gy^c^	0.97				118
CdS-Doped Glass^a^	Hybrid composites^b^	Linear: 1–40 Gy	0.85	∼20% after 2 months	33.6		132, 133

^a^ 0.4 wt. % CdS doped in glass with a composition of 70% SiO_2_, 10% Na_2_O, 10% ZnO, 6%K_2_O, 3% B_2_O_3_. ^b^Hybrid composites were composed of micromaterial doped with nanoparticles.

^c^ For protons (35 MeV) exposure.

^d^ Slope values are 0.9 and 0.93 for 6 MV and 250 kV x-rays, respectively.

^e^ Various slopes resulted from different beam qualities (kV and MV x-rays, electrons and protons).

^f^ Various slopes resulted from different beam qualities (kV and MV x-rays and protons).

^g^ Z_eff_ increases as weight % of AuNp increases.

### E. Gel dosimetry

3D gel dosimetry is a very useful tool in advanced radiation cancer therapy, especially for verifying highly sophisticated radiation dose plans prior to delivery to the patient.^134–136^ Traditional Fricke gel (FG) dosimeters, on the other hand, have two major limitations for heavy ion beam irradiation: radiation product diffusion^137,138^ and radiation sensitivity that is dependent on linear energy transfer (LET).^139^ All 3D dosimetry, besides FG dosimeter, like film, scintillation and semiconductor dosimeters, have LET-dependence as well.^140–142^ Maeyama *et al.* prepared a new Fricke gel dosimeter based on nanosynthetic hectorite (NC-FG), which is free from the aforementioned drawbacks.^143^ The obtained MRI images of that NC-FG show stable radiation sensitivities as well as negligible diffusion for 9 days after irradiation from carbon-ions. It is also revealed that the depth–dose distribution of NC-FG dosimeter agrees with that measured by an ionization chamber, indicating LET independence. Furthermore, the NC-FG can operate in both acidic and neutral conditions, and also reported that nanoclay plays an important function in the radiation-induced oxidation of ferrous ion, which was concluded from the direct proportional between the radiation sensitivity and nanoclay concentration in NC-FG.^144^ Lately, a similar gel dosimeter was prepared but without gelatin, which has three folds of radiation sensitivity, 1.8 (s^−1^ kGy^−1^), more than that of the gel that contained gelatin, 0.6 (s^−1^ kGy^−1^). When the hydrated electron scavenger N_2_O was added, a further improvement in radiation sensitivity was seen, indicating the reduction of Fe^3+^ by a hydrated electron.^145^ Another family of the gel dosimeters based on nanoclay, namely, nanoclay-based radiofluorogenic gel (NC-RFG) dosimeters using various fluorescence probes, including coumarin-3-carboxylic acid (CCA), benzoic acid (BA), terephthalic acid (TPA), trimeric acid (TMA), and pyromellitic acid (PMA),^146^ as well as dihydrorhodamine 123 (DHR 123) was proposed as a real 3D dosimeter for radiotherapy.^147^ All of these NC-RFG dosimeters showed a linear dose response within the dose range 0–5 Gy of x ray; moreover, NC-RFG-DHR 123 sensitivity had an insignificant variation throughout the post-irradiation time (37 days) and over a narrow range of the dose rate from 3.0 to 10.4 Gy/min. It was noticed that both nanoclay and radiosensitizers [Triton X-100, trichloroacetic acid (TCAA)] that were used for the leuco crystal violet (LCV) gel dosimeter contributed to the sensitivity enhancement. The diffusion of NC-RFG using DHR 123 was completely suppressed, in addition, the spatial resolution was confirmed and maintained for up to 73 days post-irradiation while autoxidation occurred. Furthermore, NC-RFG-DHR 123 was also examined in the field of brachytherapy using Ir-192 source, showing a linear response in a range of 5–100 Gy and dose rate independence within a range of 0.4–47.1 Gy/min.^148^ Mochizuki *et al.* improved some dosimetric features of this nanoclay gel sensor, such as increasing the sensitivity up to 16.2 times by adding a 1 mM TBAA (halogenide) and a 17 mM SDS (surfactant), without dose rate dependence within the range (0.77–4.67 Gy min^−1^). Because of these additives to the composition of NC-RFG-DHR 123, it has become the most sensitive gel dosimeter able to measure doses of the order of 0.1 Gy.^149^ However, the exciting light used in the dose readout by fluorometry, can produce extraneous signals with intensities that cannot be neglected in the range of a subGray dose. For that reason, new additives to NC-RFG-DHR 123 namely, N-vinylpyrrolidone (NVP) or pyridine were investigated to suppress the light sensitivity to at least 10% of ordinary conditions, resulting in a detection limit of about 0.1 Gy. This new added dispersant is assumed to stop the aggregation between rhodamine 123 (RD123) and DHR123 to prevent the oxidation of DHR123 by the adjacent photoexcited RD123.^150^ Thus, significant advantages of this nanogel over other commercial RFG dosimeters are the diffusion-free gel property and dose rate independent dosimeter for radio-therapeutic application.

Similar to this diffusion-free approach, modified cholesteryl pullulan nanogels were embedded into gel dosimeters, leading to a recent development of nanogel-incorporated 3D dosimeters.^151^ The absorbance of the prepared nanogel-incorporated Fricke hydrogel nanocomposites at 585 nm was measured by spectrophotometer while ion diffusion curves were measured by magnetic resonance imaging (MRI). The results revealed that the diffusion coefficient of nanogel-incorporated dosimeter was reduced to 0.125 ± 0.001 mm^2^ h^−1^, but the high optical dose sensitivity remains constant (0.0410 ± 0.0004 Gy^−1 ^cm^−1^). However, several aspects that are essential for any dosimeter characterization remain to be fully studied yet, for instance, dose rate and beam quality dependence, temperature stability, detection limit, and effect of the different concentration of ferric ion ligands on the dose response.

**FIG. 5.**
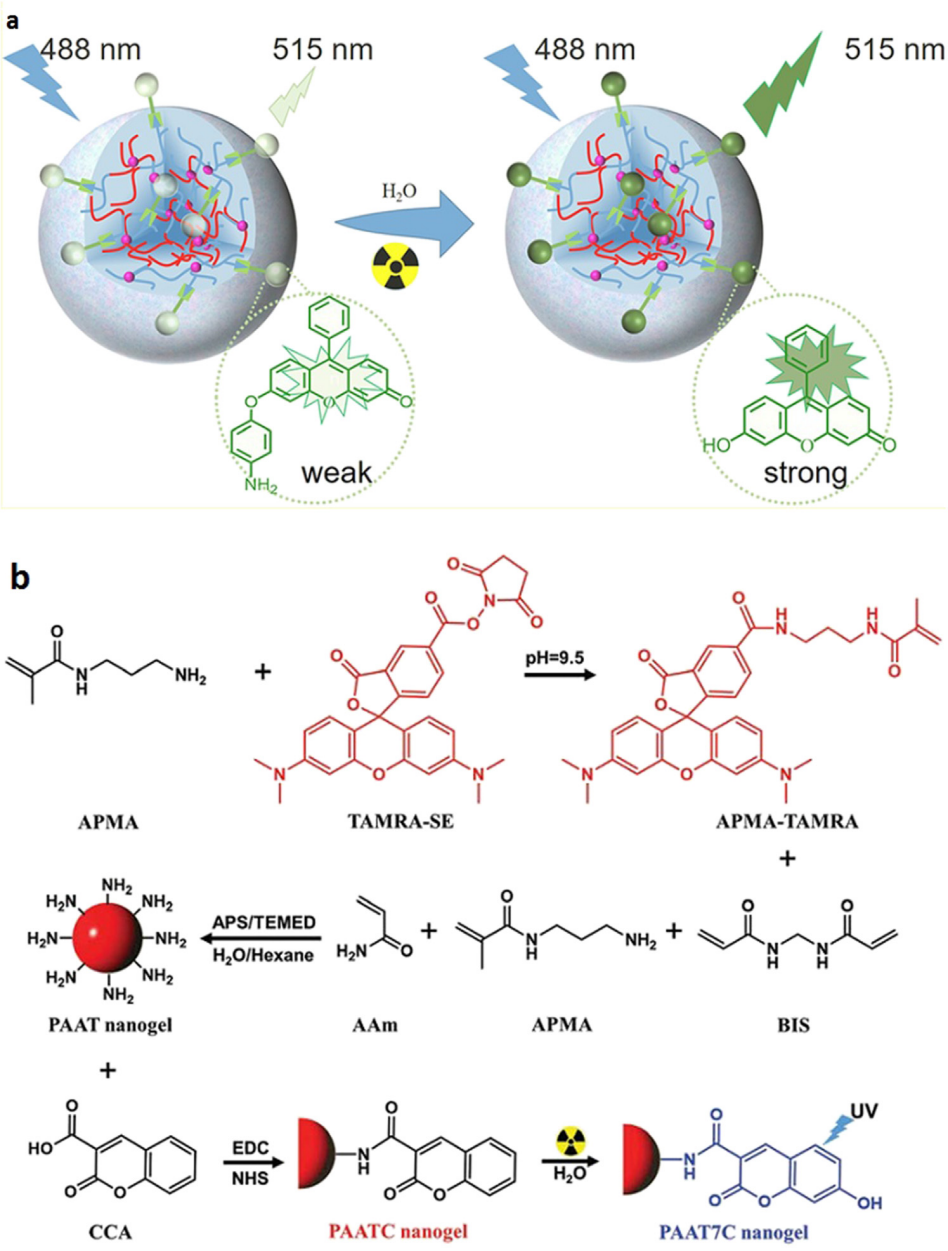
Schematic illustration of the nanogel sensing mechanism (a). Ionizing radiation converts PAA-APF with weak FL signal (on left) to PAA-F with strong FL signal (on right). Emitted FL signal of both PAA-APF and PAA-F is at 515 nm. Red-light blue strains represent the polymer chains, red small balls represent the crosslinkers, while light green balls represent weak fluorescent APF converting to strong fluorescent APF (dark green balls) by the radiolysis of water after x-ray irradiation. Reproduced with permission from Jiang *et al.*, ACS Sens. **6**, 1643 (2021). Copyright 2021 American Chemical Society.^152^ Schematic route and the concept of PAATC nanogels for ionizing radiation dose measurement (b). where N-(3-aminopropyl)methacrylamide (APMA), 5(6)-carboxytetramethylrhodamine succinimidyl ester (TAMRA-SE), N,N′-methylenebis(acrylamide) (BIS), acrylamide (AAm), ammonium persulphate (APS), N,N,N′,N′-tetramethylethylenediamine (TEMED), 1-(3-dimethylaminopropyl)-3-ethylcarbodiimide hydrochloride (EDC), N-hydroxysuccinimide (NHS), and (AAm-co-APMA-co-TAMRA)/CCA (PAATC). Reproduced with permission from Li *et al.*, Chem. Commun. **55**, 9614 (2019). Copyright 2019 Royal Society of Chemistry.^153^

X-ray dosimeters are useful for monitoring ionizing radiation exposure levels in cells and phantoms and hence for improving x-ray treatment in the clinic. In this context, a nanogel sensor based on polyacrylamide was developed, aiming to detect x-ray dose without any post-irradiation waiting time.^152^ An x-ray-responsive sensor (aminophenyl fluorescein, APF) was anchored to poly(acrylamide-co-N-(3-aminopropyl) methyl acrylamide, PAA) nanogel to create PAA-APF dosimeter. Figure 5(a) illustrates that the dose measurement is based on the change of APF to fluorescent (F) in the presence of hydroxyl radicals produced by x-ray-induced radiolysis of water molecules, converting PAA-APF to PAA-F. Therefore, more free hydroxyl radicals were produced in the solution when the x-ray dose was increased, resulting in more PAA-F nanogels, and consequently, the fluorescence (FL) intensity of resultant PAA-F nanogels at 515 nm has steadily increased. As a result, the dose of x rays may be easily monitored using fluorescence spectroscopic principles to measure the fluorescence intensity of the generated nanogel immediately after irradiation. This nanogel dosimeter has a linear dose response for x ray in a range of (0–15 Gy) with a detection limit (D_L_) of 0.5 Gy. Moreover, its dose response is stable over a wide temperature range (25–65 °C), durable over 5 weeks, and independent in energy (160 kVp and 6 MV) and dose rate (1.177 and 6 Gy/min) as well. The developer of this fluorescent sensor detected x-ray doses in A549 cancer cells as well as 3D-printed eye phantoms as a proof-of-concept. The findings revealed that this dosimeter had an ability to reliably detect doses like those utilized by radiotherapy treatment plan systems.

Li *et al.* constructed a nanogel sensor based on polyacrylamide (PAAm) for radiation dose measurement, depending on the spectral and visual colorimetric fluorescence observations.^153^ The sensor exhibits good linearity (R^2^ = 0.999), temperature stability (4–50 °C), long-term stability for about 20 days, and reproducible response with low errors, making it a promising option for x-ray-irradiated cancer cell dose detection. The colorimetric fluorescence detection of radiation dose was achieved using a PAAm-based nanogel sensor. A coumarin-3-carboxylic acid (CCA) molecule (as a sensitive fluorescent probe of ionizing radiation) and a 5(6)-carboxytetramethylrhodamine (TAMRA) molecule, as an ionizing radiation reference unit, were covalently bonded to the nanogel sensor (PAAT), producing PAATC nanogel. Under x-ray irradiation, CCA transitioned to 7-OH-CCA due to the water radiolysis, forming PAAT7C, as schemed in Fig. 5(b). Accordingly, the sensor color turns from pink to blue when radiation dose increases, and its FL signals, which are evaluated by the intensity ratio at 450 and 580 nm, rise linearly in a dose range from 0 to 20 Gy, with a 0.1 Gy detection limit as portrayed in Table VI. The main advantages of nanogel dosimeters, unlike bulk gel dosimeters, are their stable dose response over a wide range of temperature variation, long-time durability, and LET independence.

**TABLE VI.** The dosimetric properties of the prepared nanogels and nanomaterials-based gels. Slopes of these dosimeters calculated from log-log graph.

Nanogel	Detection particle	Linearity range	Slope	DL (Gy)	Post-irradiation effect	Temp. stability	Energy dependence	References
PAA-APF^a^	X-ray	Sublinear: 0–15 Gy	∼0.5	0.5	∼9% after 5 weeks	25–65°C	No	152
PAATC^a^	X-ray	Nearly linear: 0–20 Gy	0.81	0.1	Negligible up to 20 days after irradiation	4–50°C	No	153
NC-RFG-CCA^b^	X-ray	Linear: 0–50 Gy	∼0.99	NA	NA	NA	No	146
NC-RFG-TPA^b^	X-ray	Linear: 0–5 Gy	∼0.98	NA	NA	NA	No	146
NC-RFG-TMA^b^	X-ray	Linear: 0–5 Gy	∼0.93	NA	NA	NA	No	146
NC-RFG-PMA^b^	X-ray	Linear: 0–5 Gy	∼0.96	NA	NA	NA	No	146
NC-RFG-BA^b^	X-ray	Linear: 0–5 Gy	∼0.96	NA	NA	NA	No	146
NC-RFG-DHR 123^b^	MV X-ray	Linear: 0–5 Gy	∼0.9	0.1^h^	Negligible up to 37 days after irradiation	NA	No	147, 150
	γ-ray	Linear: 5–100 Gy	∼1	NA	NA	NA	No	148
AuNP-RFG-Cou^b^	X-ray	Strong sublinear: 0–2.4 Gy	∼0.27	0.3	NA	NA	No	156
CHPA-PVA-FG^c^	γ-ray	Linear: 0–30 Gy	∼0.96	NA	∼8% after 144 h.	NA	No	151
NC-FG-gelatin^d^	C^6+^ (290 MeV/n)	Strong sublinear: 0–600 Gy	∼0.33	NA	Negligible diffusion for 9 days after irradiation	NA	Moderate	144
	Ar^18+^ (500 MeV/n)	Strong sublinear: 0–800 Gy	∼0.45	NA	NA	NA	Moderate	
PVA-I_2_-SiO_2_ NP- Agarose^e^	X-ray	Linear: 0–5 Gy	∼1.02	0.5	∼50% after 5 h and the red color completely disappear within 1 day	NA	Moderate	161
nPAG-AuNP, or -BiNP	γ-ray	Strong sublinear: 0–6 Gy	0.29–0.36^e^	NA	NA	NA	Moderate	158
MAGIC-nano BiFeO_3_	γ-ray	Nearly linear: 0–16 Gy	∼0.81	NA	NA	NA	Moderate	160
	X-ray	Sublinear: 0–16 Gy	∼0.67	NA	NA	NA	Moderate	
PAGAT-BiNP	γ-ray	Strong sublinear: 1–18 Gy	∼0.31	NA	NA	NA	NA	159
NIPAM-BiNP	γ-ray		∼0.43	NA	NA	NA	NA	159
PAGAT-AgNP	γ-ray	Extreme sublinear: 6–25 Gy	∼0.17	NA	NA	NA	NA	154
MAGICA-AuNP	MV X-ray	Strong sublinear: 0–9 Gy	∼0.35	NA	NA	NA	NA	157
Gelatin-AgNP^f^	γ-ray	Nearly linear: 3–100 Gy	∼0.8	NA	Relatively stable at 6°C for a month.	dependent	NA	162

^a^ The dose response of these two nanogels were measured using intensity of fluorescence emission per unit dose.

^b^ The dose response of NC-FG-gelatin was measured using MRI techniques. The slope was calculated for the relation between R1 values and absorbed dose at Bragg peak.

^c^ The dose response of the methacrylic acid-modified cholesterol-bearing pullulan (CHPA) nanogel-incorporated Fricke hydrogels (PVA-FG) was studied by spectrophotometry.

^d^ The slopes of NC-RFGs dose response were calculated for the relation between the net fluorescence intensity and the dose.

^e^ The absorbance of the PVA-Iodine-Silica NP-Agarose complex was measured by spectrophotometer at 490 nm. ^f^The maximum absorbance at 450 nm was recorded using spectrophotometer as a function of dose.

^g^ The slopes of both Ir-192 and Co-60 dose responses of AuNP or BiNP-impregnated to nPAG were measured using MRI spectroscopy.

^h^ The detection limit for NC-RFG- DHR 123 + NVP and pyridine in the dose range of 0.016-0.47 Gy [150].

#### 1. Gel dosimetry enhancement using nanoplasmons

The well-known effect of radiosensitization of nanoplasmons as mentioned previously in the OSL and EPR sections makes the listing of the advancements in gel dosimetry due to using nanoplasmons advantageous and interesting. Here, we will focus on the improvements in gel dosimetry based on nanoplasmons as enhancer rather than that reviewed by Titus *et al.*^13^

##### a. Gel dosimetry enhancement using silver nanoparticles

To study the impact of AgNP on the sensitivity of gel dosimeters, AgNP mixed with polyacrylamide gelatin and tetrakis hydroxymethyl phosphonium chloride (PAGAT) gel dosimeters were compared to pure PAGAT.^154^ The optical density of PAGAT containing 20 nm Ag nanoparticles that varied from 1 to 3 ml in 100 ml of PAGAT, with a concentration of 3.14 mg/ml of AgNP solution, was recorded. PAGAT-AgNP gel dosimeter showed an increase in the optical densities with the dose increase from 6 to 25 Gy irradiated by Co-60. A significant improvement in the optical density-dose response with an average of 11.82% was noted for the optimal Ag nanoparticles of 2 ml. It was also observed that the DEF is dose dependent, which varied from 20% to 5%, over a dose range of 6–25 Gy, respectively. Although utilizing silver ions as a dopants requires special precautions because a redox interaction between ferrous and silver ions may occur, a signal increase which reach to 28.3% was reported by using AgNPs as a radiosensitizer in Fricke gel dosimetry.^155^

##### b. Gel dosimetry enhancement using gold nanoparticles

In 2022, a novel radio-fluorogenic agarose hydrogel enhanced with gold nanoparticles that uses coumarin (AuNP-RFG-Cou) was presented as a gel dosimeter.^156^ The effect of coumarin, agarose, and gold nanoparticles concentration and size on the fluorescence intensity was investigated in the ranges of (0.2–0.7 mM), (1–3 wt. %), (0–0.1 mM), and (30–70 nm), respectively. The obtained optimal composition was 0.1 mM gold nanoparticles, and 0.5 mM coumarin that are mixed with 3 wt. % agarose substrates. Owing to the surface plasmon resonance (SPR) effect, AuNP was added to increase the amount of hydroxyl radicals produced during the radiolysis of water. Such radicals can then react with coumarin to produce fluorescent 7-hydroxy-coumarin, leading to low-dose detection at 0–2.4 Gy and a good linear dose response. These results offer a practical method for 3D dose verification and will motivate the creation of additional radio-fluorogenic sensing hydrogels. The effect of gold nanoplasmons on another gel dosimeter, namely, MAGICA (MAGIC + agarose) was also assessed experimentally in dose enhancement for 18 MV photons. DEFs ranged from 1.014 ± 0.07 to 1.161 ± 0.15 for a concentration range (0.02–0.1 mM) of AuNP, respectively.^157^

##### c. Gel dosimetry enhancement using bismuth nanoparticles

In comparison to AuNPs, bismuth nanoparticles (BiNPs) are more desirable candidates for radiation dose amplification due to their higher atomic number, lower price, high biocompatibility, and high biodegradability. Therefore, determined DEF from the extreme sublinear dose response for 0.2 mM of both of BiNPs and AuNPs that embedded in Nomorxic polyacrylamide gel (nPAG) polymer gel at clinically applicable energies was compared.^158^ To study the energy dependence, R2 readouts of MRI scanner of the exposed samples to iridium-192 brachytherapy (380 keV) and cobalt-60 teletherapy (1.25 MeV) sources were measured. The results revealed that the mean DEF by AuNPs and BiNPs for iridium-192 source was 14.72% + 0.34 and 16.35% + 0.38, respectively, indicating that the dose enhancement by BiNPs is higher than that of AuNPs. While for the samples exposed to cobalt-60-rays, these values dropped to less than 4%, emphasizing the moderate energy dependence of DEF.

Additionally, the DEF using BiNP in both of poly acrylamide gelatin tetrakis hydroxymethyl phosphonium chloride (PAGAT) and nisopropylacrylamide (NIPAM) was examined.^159^ It was evaluated by measuring the absorbance of doped gels with BiNP particles at concentrations from 0.5 to 2.5 mM as a function of Co-60 Gamma doses. A strong sublinear dose response for both BiNP-based gels was reported in a dose range of 1–18 Gy with good correlation coefficients to some extent, with an average of about 0.95. The maximum DEF was for a 0.5 mM BiNP concentration for both PAGAT and NIPAM, and its value was 1.34 and 1.18, respectively.

Rajaee *et al.* proved also the feasibility of bismuth ferrite nanoparticles (BiFeO_3_)-incorporated methacrylic and ascorbic acid in gelatin initiated by copper (MAGIC) gel dosimeter as a magnetic localized dose enhancement agent.^160^ Using MRI, R2 data of irradiated gel with and without 100 nm BiFeO_3_ nanoparticles were compared as a function of x- and gamma ray doses of up to 16 Gy. The calculated DEF for 0.5 mg/ml concentration of BiFeO_3_ nanoparticles was 2 and 1.6 at 160 keV x ray and 380 keV from Ir-192 source, respectively.

### F. Colorimetry

Through most radiation dosimetry systems there is a need for advanced technical fabrication which leads to higher cost systems and in many cases skilled operators for reading them out.^163,164^ A colorimetric nanosensor dosimeter based on hydrogel was developed for x ray and proton beams,^165,166^ using gold nanoparticles within the gel matrix by the reduction of gold ions in both qualities, with linearity range of 0–3 and 0.5 Gy detection limit. Similar detection systems based on nanoplasmonic colorimetric materials have demonstrated substantial advantages over conventional sensors, including higher stability, biocompatibility, sensitivity, and importantly they are relatively inexpensive.^167,168^ The applicability of the prepared radiation-responsive gel nanosensors to be used as a platform technology for ionizing radiation detection in three different form factors was recently studied, with a view toward diverse clinical use.^169^ Ionizing radiation reduces monovalent gold salts in this approach, resulting in the formation of gold nanoparticles within gel matrix formulated in a variety of morphologies such as one-dimensional (1D) needles and two-dimensional (2D) area inserts for multiple modes of brachytherapy, as well as 3D volumetric dose distribution in tissue phantoms. The produced gold nanoparticles can be detected utilizing various but independent means of readout including visual and photothermal detection, which further boosts the adaptability of this approach. Linear dose response in readout has been demonstrated, allowing for simple calibration.

**FIG. 6.**
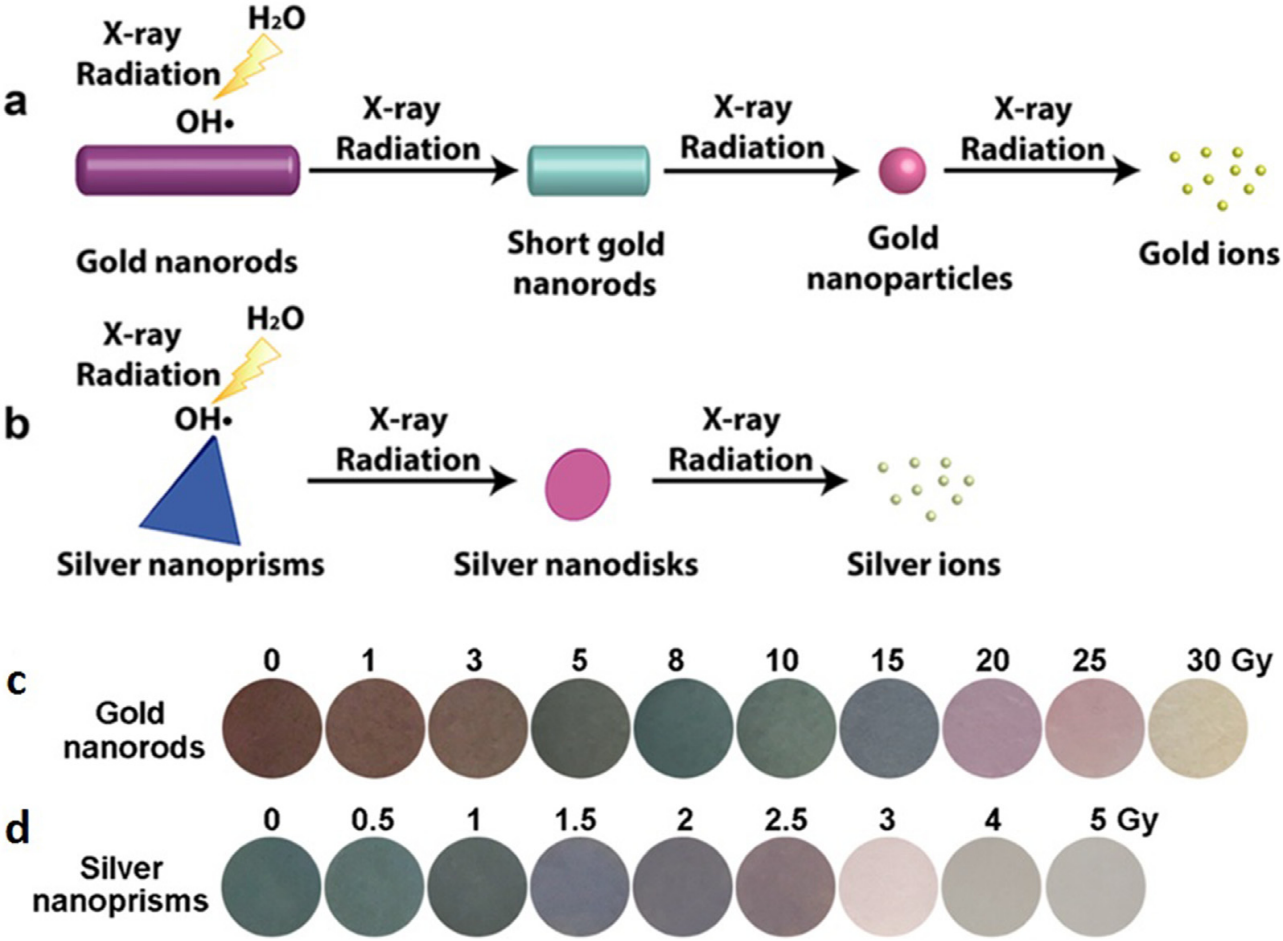
Mechanism illustration of radiation-based etching of Gold nanorods (AuNRs) converted to gold ions through short gold nanorods and gold nanoparticles depending on the radiation dose (a) and Silver nanoprisms (AgNPRs) to silver nanodisks then silver ions depending on the radiation dose (b). Color change of irradiated paper-based colorimetric assays for a gold nanorods shifts from burgundy at 0 Gy, to pale yellow at 30 Gy (c) and a silver nanoprisms assay shifts from blue at 0 Gy to colorless at 5 Gy (d). These assays are largely stable in the absence of radiation. Reproduced with permission from Tao *et al.*, ACS Appl. Mater. Interfaces **12**, 22499 (2020). Copyright 2020 American Chemical Society.^174^

Furthermore, the special characteristics of gold nanorods (AuNRs) and silver nanoprisms (AgNPRs) coated with cetyltrimethylammonium bromide (CTAB), such as ease production and modification, with tunable optical properties, and superb temperature sensitivity and solubility, improves their attraction.^170–173^ Tao *et al.* established a new radiation nanosensor in a different way via etching of gold nanoparticles and silver nanodiscs to their corresponding ions by radiation induced hydroxyl radicals as illustrated in Figs. 6(a) and 6(b), resulting in reproducible wavelength shift (i.e., color changes).^174^ This colorimetric sensor enabled the detection of radiation doses visually in a range of 0.5 to 20 Gy with high sensitivity and reproducibility. The AuNRs color varied from burgundy at zero dose to pale yellow at 30 Gy while AgNPRs changed from blue for unirradiated to colorless at 5 Gy as shown in Fig. 6(c) and 6(d). These nanosensors based on AuNR and AgNPR have been designed as a paper test, which present new radiation dosimetry tool with prospective applications in qualitative assessment of delivery.

This trend of detection and classifying of the irradiated regions by a colorimetric gel nanosensor was demonstrated by Pushpavanam *et al.*.^175^ They studied the effective factors on the formation conditions including gold salt and gel pore sizes, surfactant concentrations, and administrating the quenching agent to optimize the nanosensor performance. The results revealed that the efficiency of the nanosensor was enhanced by controlling both the size and allocation of pores inside the gel substrate. This gel nanosensor was used to visualize a variety of basic and complicated dose modes in fractionated clinical radiotherapy, demonstrating its ability to capture dose profiles. The effectiveness of this gel nanosensor as compared to the Gafchromic EBT3 films was assessed. They noted that the gel nanosensor technology was suitable for determining complicated topographical distribution of dose profiles in clinical radiotherapy. This was due to the ease of preparation, simple operation procedures, fast and stable readout, and colorimetric detection. One of the main issues of this type of dosimeters is the high combined uncertainty in the dose assessment. The summary of colorimetric properties of the prepared nanosensors is demonstrated in Table VII.

**TABLE VII.** The colorimetric properties of the prepared nanosensors. Slopes of these dosimeters are calculated from log-log graph. All dosimeters were measured using UV-Vis spectrophotometer after irradiation to x-ray, except for Th-SINAP-100 and C6H5O7Na3-AgNP were irradiated to γ-ray.

Nano-sensors	Linearity range	Slope	DL (Gy)	Post-irradiation effect	Energy dependence	References
Agarose/AuNP/ C_12_TAB^a^	Nearly linear: 0–3 Gy	0.83	0.5	NA	Negligible	166
	Linear: 0–3 Gy^e^	∼1.03	0.5	Stable up to 4 h	Negligible	165
Agarose/AuNP/ C_14_TAB^a^	Linear: 1–4 Gy	∼1.05	2	Negligible during a week	Negligible	175
	Linear: 0–3 Gy^e^	∼1.09	0.5	Stable up to 4 h	Negligible	165
Agarose/10 mM AuNP/C_16_TAB^b^	Supralinear: 0.5–2 Gy^f^	∼1.5	0.5	Negligible up to 7 h	Negligible	179
	Supralinear: 5–37 Gy^g^	∼1.5	5			
Th-SINAP-100^c^	Strong Sublinear: 1–10 kGy	∼0.34	NA	Negligible up to 48 h	Very Strong	178
	Very strong Sublinear: 10–80 kGy	∼0.22	NA			
AuNP/CTAB	Nearly linear: 1–20 Gy	∼0.83	1	Peak shift is time dep. up to 2 h from irradiation^h^	NA	174
AgNP/CTAB	Nearly linear: 0.3–3 Gy	∼0.85	0.5	Peak shift is time dep. up to 0.5 h from irradiation^i^	NA	174
RANGs	Linear: 0–6 Gy	∼1	0.8	Negligible during a week	NA	176
C_6_H_5_O_7_Na_3_-AgNP^d^	Sublinear: 2–120 Gy	∼0.77	2	Stable for 5 days	NA	177

^a^ The slope was calculated from the maximum absorbance of these dosimeters after 1 h irradiation to x-ray.

^b^ The absorbance measurement of this nanosensor was done after 2 h irradiation.

^c^ The slope was linearly fitted from the relation between RGB integer (green) and γ-ray dose.

^d^ The absorbance of 0.1% of sodium citrate mixed with 1 mM of AgNO_3_ at 420 nm.

^e^ For proton doses in a unit of (Gy_RBE_)

^f^ For C_16_TAB with a 2 mM concentration.

^g^ For C_16_TAB with 10 and 20 mM concentrations.

^h^ Wavelength shift of absorption spectrum maximum reaches 230 nm after 2 h, stabile to 160 h.

^i^ Wavelength shift of absorption spectrum maximum reaches 120 nm after 0.5 h, stabile to 160 h.

A further new colorimetric approach for radiation detection was proposed recently, based upon gold nanoparticles being shaped by amino acids after being exposed to ionizing radiation.^176^ A library containing 32 amino acids (20 natural, 7 artificial, and 5 optical isomers) was screened to test their ability to enhance the creation of gold nanoparticles when irradiated to x rays or photons, which was observed as an increase in usual absorbance wavelengths. It was found that aspartic acid, leucine, phenylalanine, threonine, and valine, each tend to template AuNP formation after exposure to ionizing radiation. The role of these amino and carboxy moieties in determining NP formation of phenylalanine was revealed by nuclear magnetic resonance (NMR) spectroscopy. Radiation responsive amino acid nanosensor gels (RANGs) based on phenylalanine and tryptophan were fabricated based on the potency for interactions between the aromatic phenyl group in phenylalanine and the indole moiety in tryptophan. RANGs demonstrated a linear response of the absorbance at a peak wavelength of 530 nm with x-ray doses between 1 and 6 Gy with a 0.8 Gy detection limit and relatively stable behavior over a week. Although RANGs stand out for their biocompatibility, quick response, ease of fabrication and operation, accuracy, and long-term readout, they still need further optimization to realize their ultimate sensitivity and precision.

A new state of colorimetry system based on Ag nanoparticles was established for dosimetry purposes.^177^ Two different concentrations of sodium citrate (1 and 0.1% C_6_H_5_O_7_Na_3_) and silver nitrate (1 mM AgNO_3_) were prepared as precursors for AgNPs in an aqueous colloidal solution and were examined in the dose range from 0.5 to 120 Gy, changing from colorless (unirradiated) to yellow (irradiated). Spectrophotometer absorbance results indicated that synthesis of AgNPs occurred in 0.1% concentration of sodium citrate solution induced by gamma radiation occurred starting from 2 Gy. It was also found that this liquid dosimeter has a sublinear relation for the dose response study with stable readouts for 5 days post-irradiation.

In unprecedented behavior of all nanomaterials-based colorimetric dosimeters, Lu *et al.* presented a dual-module (color and luminescence) photochromic metalorganic nanocluster, [Th_6_(OH)_4_(O)_4_(H_2_O)_6_](TPC)_8_(HCOO)_4_·4DMF·H_2_O (Th-SINAP-100, TPC = 2,2′:6′,2′′-terpyridine-4′-carboxylate, DMF = dimethylformamide), for ultrahigh beta and gamma ray dosimetry.^178^ Qualitative ionizing radiation detection is easily achieved by the photochromic change from purple to yellow. An on-site quantitative platform for visible detection of dose over a wide range was made possible by the color shift and, more importantly, the visible color transition of luminescence from blue, to cyan, and to green in response to accumulated radiation doses (1–80 kGy). Using a digital camera and graphic software, the response of radiation dose could be monitored by the change of the red, green, and blue (RGB) parameters of the PL collected from the Th-SINAP-100. Furthermore, this colorimetric dosimeter was loaded on polyvinylidene fluoride (PVDF) polymer producing a flexible strip, like a commonly used pH strip. The obvious drawback of these is the very high Z_eff_ (≈62) which indicates that it would likely be an extremely energy-dependent dosimeter. However, Th-SINAP-100 is particularly well suited for ultrahigh dose (kGy level) monitoring in a variety of fields, including synchrotron radiation, medical sterilization, food irradiation, and radiodegradation.

In summary, these nanocolorimetric dosimeters have their strongest advantages in being lower cost options for a visible, simple, and user-friendly stable indicators of radiation dose, which could be optically tuned based upon the materials. These are all good features and make them attractive for specific applications where ease of use and cost dominate the decision making, perhaps with less attention to quantifying the exact dose vs verification that dose has been delivered.

### G. Polymer dosimeters

A multicompartment 3D radiation dosimeter based on polymer nanocomposite was established and demonstrated how its unique features may be used to exploit the interaction of radiation with metal nanoparticle experimentally in 3D.^180^ This polymer nanocomposite dosimeter, termed sensitivity modulated advanced radiation therapy (SMART), was developed as a tissue equivalent (TE) material composed of mixed clear polyurethane resin, halocarbon radical initiator, and leuco malachite green (LMG) dye at a concentration of 0.5 mM of AuNPs (∼50 nm). The optical absorption of this dosimeter was acquired at 633 nm wavelength. The obtained DEFs in the range of 0–30 Gy for 100 kV and 6 MV x rays were 1.77 and 1.11, respectively. The inter-batch variability in dosimeters with AuNPs was less than 3.5% and the intra-batch variability was insignificant (<0.5%). The overall results were consistent with previous *in vivo* and *in vitro* experiments as well as theoretical modeling.^181,182^ The importance of the SMART dosimeter described in this work was its capability to provide extensive experimental 3D imaging of nanoparticle-enhanced radiation treatment. Because the TE composition of the SMART dosimeter has the same Z_eff_ as water, it can be used to simulate radiation–tissue interactions.

The efficiency of two different types of bismuth nanoparticles as agents for enhancing polymer dosimeter sensitivity has been demonstrated experimentally. Spectrophotometric phantom cuvettes were manufactured by doping with AuNPs and Bi-based nanoparticles, forming 3D phantoms. The net optical density, the change in the absorbance intensity at 632 nm wavelength between the irradiated phantom and unirradiated one, showed a linear relation with the absorbed dose. The attained enhancement of the radiation dose by Bi_2_O_3_-NPs and Bi_2_S_3_-NPs was compared to that of the well-researched AuNPs throughout a dose range of 1–20 Gy. For 100 kV energy, the DEFs obtained for 50 nm of Bi_2_O_3_-NPs and AuNPs with a concentration of 0.5 mM for both that were incorporated into polymer dosimeters were 1.90 and 1.77, respectively. While at 150 kV x ray, the DEFs of 5 nm Bi_2_S_3_-NPs and AuNPs, with a concentration of 0.25 mM for both, were calculated to be 1.38 and 1.51, respectively. The results revealed that BiNP materials could enhance the sensitivity of polymer dosimeters. In comparison to kilovoltage energy, the DEFs due to all the nanoparticles under investigation for 6 MV x rays were lower by around 15%. All NPs in this study had a direct proportional effect with their concentrations from 0.1 to 0.5 mM. Given that Bi is one of the least expensive heavy metals, is easy to create compounds in nanophase, and has low toxicity, it should be further investigated to fully understand the enhancement mechanism.^183^ The nanopolymeric dosimeters can be exploited in the volumetric dosimetry for (1) high sensitivity, (2) 3D spatial resolution, (3) superior intra-batch stability, (4) good post-irradiation stability, and (5) reasonable tissue equivalency.

Generally, the incorporation of plasmonic nanoparticles, such as silver, gold or bismuth into the commonly used microstructured OSL, ESR, gel, colorimetric, or polymer dosimeters improves their sensitivity. Although much research has addressed the enhancement of such dosimeters by plasmons, some dosimetric properties still need to be investigated to point out the possible fields of their use, such as the post-irradiation effect, reusability, thermal stability, and cost per dosimeter.

### H. Ionization chambers

Ionization chambers (ICs) are regarded as the most directly trusted tools for dosimetry due to their high accuracy, reliability, practicality, and ability to measure absolute ionization values. However, their size can minimize their value due to limited spatial resolution and thickness. They are rarely used for *in vivo* dosimetry and so many of the previously mentioned tools are developed for this. Relatively few developments in ICs have focused on nanomaterials; however, Funaro *et al.* proposed new real time radiation dosimeter having electrodes based on vertically aligned multiwall carbon nanotubes (MWCNTs) or copper covered with graphene.^184^ They investigated the efficiency of the charge collection for these detectors and compared them with conventional electrode materials (copper, aluminum, and silicon). At a standard bias voltage, the suggested dosimeters had excellent linear response to radiation dose and gathered more charge than their compared reference dosimeters. This development could allow for future miniaturized ICs. Furthermore, a MWCNT-based IC has the highest charge collection efficiency and can perform at lower bias voltages, making them applicable for most *in vivo* dosimetry applications where possible. In comparison to reference dosimeters, graphene-based ICs perform better (i.e., collect more charge) at a standard bias voltage (310 V). However, as compared to a reference detector, the charge collection efficiency decreases as the bias voltage decreases, owing to graphene's semiconducting characteristics. Further analysis and development of this area remain to be studied.

## III. SUMMARY

**FIG. 7.**
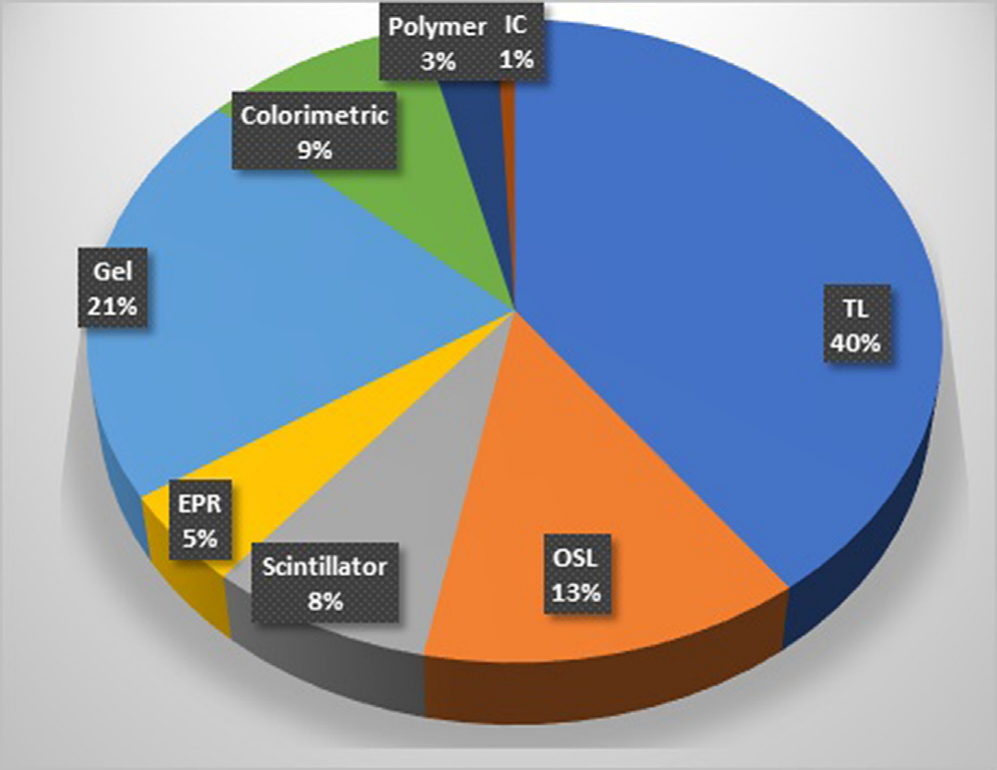
The fraction of the 130 nanomaterial-based dosimeters studied here are illustrated, brokendown into the readout methods or dosimetry tool. The largest share is for TL dosimeters (about 40%), while the lowest one is for the ionization chambers (about 1%), illustrating where the current state of nanomaterials is having its relative potential impacts in dosimetry.

Finally, the categories of the radiation fields that have already been investigated and discussed in this comprehensive review of the dosimetric study using various types of nanomaterials-based dosimeters are classified in Table VIII, according to the studied range of the dose or dose rate or the radiation sources. As is evident in this table, the research in numerous areas of radiation dosimetry, particularly in the neutron field, using nanotechnology has not been covered, leaving open the possibility for future studies or advancement in the current capabilities of the dosimeters. The application of nanotechnology in radiotherapy, on the other hand, is one of the most searched disciplines to accomplish the accuracy in dose measurement of the continuous advancement in cancer therapy plans. Of the approximately 130 dosimeters reviewed in this paper, based on nanomaterials, their breakdown based upon readout methods or dosimetry tool is illustrated and categorized in Fig. 7. It is clear that the highest percentage of the newly manufactured dosimeters based on nanomaterials are those using the TL technique (about 40%) while the lowest percentage is that for ICs (about 1%). This could be because TL dosimeters are easily prepared, know-how is widely available, and the readout uses a low-cost apparatus, whereas using nanotechnology in ICs requires experienced personnel who are not available to multiple research groups, as well as precise and expensive fabrication tools.

**TABLE VIII.** Summary of radiation field categories that were studied using different types of currently prepared dosimeters based on nanomaterials. The range of the dose levels are in parentheses.

Nanomaterials-based dosimeters	Environmental dosimetry (up to 1 mGy)	Personal and diagnostic dosimetry (1–100 mGy)	Radiotherapy dosimetry (0.1–100 Gy)	High dosimetry (100–1000 Gy)	Ultra-high dosimetry (greater than 1 kGy)	Charged particles dosimetry	Neutron dosimetry
TL	57	44, 50, 57, and 66	44, 46–48, 50, 55, 61–63, and 67	44–48, 50, 55, 58, 61–63, and 67	46, 55, 58–60, 62, 63, and 67	33, 36, 37, 41, 66, and 67	⋯
OSL	⋯	50, 66, 79, 88, and 93	50, 66, 76, 79, 92, and 93	50 and 93	⋯	66, 76, and 79	⋯
Scintillator	104 and 114	98, 99, 104, 107, 110, and 112–114	107 and 113–115	110 and 113	⋯	110	⋯
EPR	⋯	⋯	117, 126, 127, 130–133	117	⋯	118	⋯
Gel	⋯	150, 153, and 156	146–148, 150–154, 156–159, 161, and 162	148	⋯	144 and 145	⋯
Colorimetric	⋯	⋯	166, 169, 174–177, and 179	⋯	178	165	⋯
Polymer	⋯	180 and 183	180 and 183	⋯	⋯	⋯	⋯
Ionization chamber	⋯	⋯	184	⋯	⋯	⋯	⋯

## IV. CONCLUSIONS

This review is focused on the improvements in dosimetry systems that could be achieved by the creation of new nanomaterials that would contribute to the detection of different types or ranges of ionizing radiation. The most recent research reviewed on direct and passive dosimetry systems included polymer, gel, TL, ESR, and OSL dosimeters, in addition to scintillators, colorimetric nanosensors, and ionization chambers as platforms for radiation sensing. The nanophase composition of these systems, which have luminescence phenomena except RPL (i.e., TL and OSL), show properties that indicate that there is excellent potential for the widening of the linearity range of the absorbed doses due to their high radiation resistance. This range widening might be due to the self-absorption of the generated photons by stimulated deep traps in the microcrystals being more significant than that of nanostructured dosimeters. Moreover, the intensities of the TL or OSL curves for nano-sulfates are almost stable over a long period of time in comparison to bulk sulfates. Contrary TL, it seems that OSL dosimeters based on nanomaterials could have greater sensitivity than that of their microcounterparts, which might be attributed to the higher absorption efficiency of the laser by NPs than it is by bulk material. Although RPLs have many advantages as compared to other luminescence phenomena that used for radiation measurements and dosimetry. The material options are limited, and many applications tested to date have only taken conventional materials into account, but nanoscale properties are sometimes radically different than their microscale or macroscale counterparts. Recent research studies, reviewed here, have greatly expanded the material options available, and the chosen materials may have possibility for replacement of current materials and introduction of new applications for RPL. Like TLDs and OSLs, improvements in the dosimetric features of RPL materials have been shown, and so new dosimeters exploiting these should be expected. Using EPR technique, nano-sulfates can also enhance the dosimetry, in contrast to their bulk form, due to their high sensitivity, wider dynamic range, and longer lifetime stability, as well as their ability to work in a humid environment.

Nanoscintillators have several benefits over bulk typical scintillating materials, including visible emission, tunable bandgap, efficient photon counting, high photon yield, and effective operation at ambient temperature. Perovskite nanocrystals have promising properties, such as an extreme PL quantum yield, high-performance inexpensive x-ray detectors with high resolution, sensitivity, and stability, allowing them to be introduced as novel nanoscintillators. To overcome the energy dependence of perovskite, plastic scintillators based on perovskite as a kind of inorganic–organic nanoscintillators, keeping the tremendous advantages of perovskite, were fabricated to be applicable in nuclear detection, medical imaging, and physics of high-energy. On the other hand, the synthesis of quantum dots to obtain high optical quality particles with well-defined structure requires a mix of experience and intensive research.

It is worthwhile to mention that coupling certain low-density dosimeters with high Z_eff_ nanoparticle plasmons, for instance, silver, gold, and bismuth, is another way of enhancing their sensitivity, particularly in OSL, EPR, gel, polymer, and colorimetry dosimetry systems. Such doped tissue equivalent dosimeters mimic the radiotherapy energy dissipation behavior and can be used to accurately determine the radiation dose enhancement factor. The advantages and obstacles of translating these technologies to clinical and industrial uses were also discussed. One of the main drawbacks of a potential dosimeter containing high Z_eff_ nanomaterials in radiation dosimetry applications is the response discrepancy for different beam qualities. This can be overcome by distributing the nanophosphor in a transparent plastic or liquid with a low Z_eff_ and making the dosimeter tissue equivalent. These properties of dosimetry systems that contain nanomaterials show key improvements that will better satisfy the requirements for improved radiation detection in several industrial and medical applications.

The main advantages of nanogel dosimeters, unlike bulk gel dosimeters, are their stable dose response over a wide range of temperature variation (reaching 45 °C), long-term durability, and LET independence. In addition to the high combined uncertainty in the radiation dose assessment using the gel nanosensors, there is another drawback, which is the shorter linearity range (less than an order of magnitude in some cases). Whereas, the detection systems based on nanoplasmonic colorimetric materials have demonstrated substantial advantages over conventional sensors, including higher stability, simplicity, biocompatibility, sensitivity, and importantly they are relatively inexpensive. An on-site quantitative platform for visible detection of dose over a wide range was made possible by the color shift and, more importantly, the visible color transition of luminescence in response to accumulated radiation doses. Regarding nanopolymeric dosimeters, the key benefits of their use for volumetric dosimetry are their outstanding sensitivity, 3D spatial resolution, improved intra-batch stability, good post-irradiation stability, and adequate tissue equivalency.

Frequent establishment of new nanomaterials-based dosimeters in some dosimetry systems like TL and gel rather than others like IC could be attributed to the fact that the most frequently prepared dosimeters are easy to prepare, know-how is widely available, and readouts use low-cost devices. While the use of nanotechnology in the most complicated systems requires experienced personnel that is not available to multiple research groups as well as precision and cost-effective manufacturing tools. The solid-state dosimetry systems based on nanomaterials like TL, OSL, EPR, and nanoscintillators can be fabricated easily to some extent in nanophase or coupled with nanoplasmons, in contrast to organic dosimeters like gel, polymer, and colorimetry, all of them were doped by nano-inorganic materials as radio-sensitized. The causes behind this are unknown, although they could be because pure organic materials have a negligible radiation effect or exhibit no variation when compared to their microcounterparts.

In future applications, nanotechnology offers some solutions for problems that have not been fully explored to date, such as multi-energy and multi-particulate radiation sources. Some examples of this are that the use of nanotechnology in radiation dosimetry for neutron or environmental fields or using ICs in various dose ranges have not been fully examined but could be beneficial. Additionally, there are substantial advances in Monte Carlo modeling of multiscale phenomena, and advanced methods to include nanoscale surface effects into radiation transport Monte Carlo studies will be highly beneficial to better understand these newer complex materials as radiation detectors. On the other hand, the use of nanotechnology in radiotherapy is one of the most demanded fields to achieve accuracy in dose measurement for ongoing developments in cancer therapy regimes. The material developments reviewed here provide the initial background material for further testing and computational analysis of features that will aid research or improvements in current capabilities of complex radiation dosimetry needs.

## Data Availability

The data that support the findings of this study are available from the corresponding author upon reasonable request.
